# New Volatile Perfluorinated Amidine–Carboxylate Copper(II) Complexes as Promising Precursors in CVD and FEBID Methods

**DOI:** 10.3390/ma14123145

**Published:** 2021-06-08

**Authors:** Katarzyna Madajska, Iwona Barbara Szymańska

**Affiliations:** Department of Chemistry, Nicolaus Copernicus University in Toruń, Gagarina 7, 87-100 Toruń, Poland; 502533@doktorant.umk.pl

**Keywords:** chemical vapour deposition, focused electron beam induced deposition, volatile compounds, volatility study, nanomaterials, electron interactions, copper fluoride, thermal properties

## Abstract

In the present study, we have synthesised and characterised newly copper(II) complexes with the general formula [Cu_2_(NH_2_(NH=)CC_2_F_5_)_2_(µ–O_2_CR_F_)_4_], where R_F_ = CF_3_, C_2_F_5_, C_3_F_7_, C_4_F_9_. Infrared spectroscopy, mass spectrometry with electron ionisation (EI MS), and density-functional theory (DFT) calculations were used to confirm compounds’ composition and structure. The volatility of the compounds was studied using thermal analysis (TGA), EI MS mass spectrometry, variable temperature infrared spectroscopy (VT IR), and sublimation experiments. Research has revealed that these compounds are the source of metal carriers in the gas phase. The thermal decomposition mechanism over reduced pressure was proposed. TGA studies demonstrated that copper transfer to the gaseous phase occurs even at atmospheric pressure. Two selected complexes [Cu_2_(NH_2_(NH=)CC_2_F_5_)_2_(µ–O_2_CC_2_F_5_)_4_] and [Cu_2_(NH_2_(NH=)CC_2_F_5_)_2_(µ–O_2_CC_3_F_7_)_4_] were successful used as chemical vapour deposition precursors. Copper films were deposited with an evaporation temperature of 393 K and 453 K, respectively, and a decomposition temperature in the range of 573–633 K without the use of hydrogen. The microscopic observations made to investigate the interaction of the [Cu_2_(NH_2_(NH=)CC_2_F_5_)_2_(µ–O_2_CC_2_F_5_)_4_] with the electron beam showed that the ligands are completely lost under transmission electron microscopy analysis conditions (200 keV), and the final product is copper(II) fluoride. In contrast, the beam energy in scanning electron microscopy (20 keV) was insufficient to break all coordination bonds. It was shown that the Cu-O bond is more sensitive to the electron beam than the Cu-N bond.

## 1. Introduction

Nowadays, copper compounds are used, for example, to design hybrid organic-inorganic materials [1,2,3], anticancer agents [4,5], and sensors [6,7]. Additionally, copper complexes are used as model compounds and tested as antimicrobial agents [8,9,10].

On the other hand, copper nanostructures are used, for example, in electronics [11,12,13], plasmonics [14,15], catalysis [16,17,18], and as antibacterial agents [19,20]. These structures can be obtained by gas-assisted methods such as chemical vapour deposition (CVD) [21,22,23] and focused electron beam induced deposition (FEBID) [24,25,26]. CVD is a process where one or more volatile precursors are transported in the vapour carrier gas to the reactor chamber, where they decompose on a heated substrate and deposit a solid material [27]. In FEBID, the precursor is supplied without the use of carrier gas, then the adsorbed compound is dissociated in the focus of an electron beam provided by a scanning or transmission electron microscope (SEM/TEM) [28]. For the FEBID deposition of metals, CVD precursors are often tested. However, in addition to being sufficiently volatile, the compound must also decompose under the influence of an electron beam [29]. In copper FEBID, several complexes previously used as CVD precursors, such as [Cu(hfac)(VTMS)], [Cu(hfac)(MHY)], and [Cu(hfac)(DMB)] of Cu(I); [Cu(tbaoac)_2_], [Cu(hfac)_2_]·H_2_O, and most recently [Cu_2_(µ-O_2_CC_2_F_5_)_4_] of Cu(II), were applied, but the purity (10–26 at.%) of the formed deposits remains a problem [30,31,32,33].

There are no literature data about the synthesis of Cu(II) perfluorinated amidinate and heteroleptic amidine-carboxylate compounds and research on their volatility to the best of our knowledge. In the available literature, only silver(I) and mercury(II) compounds with perfluorinated amidinates are known: [Ag_2_((NH)_2_CCF_3_)_2_], [Ag_2_((NH)_2_CC_2_F_5_)_2_], and [Hg((NH)_2_CC_2_F_5_)_2_], which however, have not been tested for use in vapor deposition methods [34]. Only copper(I) amidinate complexes with non-fluorinated ligands of the general formula [Cu_2_(AMD)_2_], known from the ALD (atomic layer deposition) process [35], have been tested in the CVD process. For example, when [Cu_2_(i-Pr-MeAMD)_2_] was vaporised at 368 K and hydrogen was used as a reducing gas, pure copper films were obtained in the temperature range 473–573 K [36]. Using the same precursor, copper films were deposited on steel, silicon, and SiO_2_/Si substrates at 473–623 K, at a total pressure of 1333 Pa with hydrogen as a reactive gas [37]. On the other hand, in 2019, (*N,N′*-di-sec-butylacetamidinato)copper(I) was used to produce Cu_2_O thin films in a water assistance process in a low-temperature range of 398−473 K [38]. In all of the above-mentioned CVD processes, although copper(I) compounds were used, which are often spontaneously disproportionate to copper(0), it was necessary to use hydrogen as a reducing agent. Furthermore, both the synthesis and the manipulation of the used compounds should be carried out under inert gas [39].

It is known that fluorinated ligands favour thermal stability and vapour pressure [40]. Copper CVD precursors containing perfluorinated carboxylates and N-donor ligands are known. [Cu_2_(^t^BuNH_2_)_2_(μ-O_2_CR_F_)_4_] (R_F_ = C_n_F_2n+1_, *n* = 1−6) were the source of volatile copper species: [Cu_2_(^t^BuNH_2_)_2_(μ-O_2_CR_F_)_4_] and [Cu_2_(O_2_CR_F_)_2_], which were transported in the gas phase in the temperature range of 473–533 K. Metallic copper films were obtained between 623 K and 733 K without an additional reducing agent [41,42].

On the other hand, in their 2008 paper, Brown et al. reported the amidinate–carboxylate complexes of dimolybdenium(II) and ditungsten(II) [M_2_(O_2_CMe)_2_((N^i^Pr)_2_CR)_2_] obtained from the reaction of the [M_2_(O_2_CMe)_4_] and the lithium amidinates Li{(N^i^Pr)_2_CR}, where M = Mo, W and R = Me or M = Mo and R = –C≡C^t^Bu, –C≡CPh, and –C≡C–Fc (Fc = 1-ferrocenyl) [43]. In other studies [Mo(amidinate)(O_2_CR)_3_] or [W_2_(amidinate)_2_(O_2_CR)_2_] complexes with bridging amidinate were obtained in reaction of [M_2_(O_2_CR)_4_] (M = Mo, W, R = CF_3_) with one and two equivalents of Li{(N-2,6-^i^Pr_2_C_6_H_3_)_2_CH}(THF)_2_, respectively [44]. However, in the mechanochemical reaction of unsymmetrical *N,N′*-diarylformamidine ligands (Ar-amidine), for example, *N*-(2-methoxyphenyl)-*N′*-2,6-dichorophenyl)-formamidine with copper(II) acetate and zinc(II) acetate, paddle wheel complexes of general formula [Zn_2_(Ar-amidine)_2_(OAc)_4_] and [Cu_2_(Ar-amidine)_2_(OAc)_4_] were synthesised. In the obtained complexes, the N-coordinated ligand in the axial position remained protonated [45].

We decided to synthesise compounds containing a perfluorinated carboxylate and a perfluorinated amidine as a secondary ligand in the axial position based on the previously mentioned literature results. We wanted to investigate how the volatility and thermal stability of the compound would be affected by introducing the perfluorinated amidine instead of the previously used amine with bulky group. In this paper, we discuss the synthesis of novel copper(II) complexes with the general formula [Cu_2_(NH_2_(NH=)CC_2_F_5_)_2_(O_2_CR_F_)_4_], where R_F_ = CF_3_, C_2_F_5_, C_3_F_7_, C_4_F_9_. For simplicity, the abbreviation AMDH for C_2_F_5_C(=NH)NH_2_ is used in this work. We also describe the structural characteristics and thermal properties of the new compounds. Importantly, we examined the volatility of the compounds and their interactions with an electron beam. We selected the most promising compounds and used them in preliminary chemical vapour deposition experiments based on volatility studies.

## 2. Materials and Methods

CuCO_3_·Cu(OH)_2_ (>95%), anhydrous acetonitrile (99.8%), CF_3_COOH (99%), and RCOOH (R = C_n_F_2n+1_; *n* = 2–4; 97–98%) were purchased from Sigma Aldrich (Saint Louis, MO, USA) and used as received. Pentafluoropropylamidine C_2_F_5_C(=NH)NH_2_ (AMDH) (98.7%) was purchased from Apollo Scientific (Stockport, UK) and used as received. Copper(II) carboxylates [Cu_2_(µ-O_2_CR_F_)_4_], where R_F_ = C_n_F_2n+1_, *n* = 1–4, were prepared as earlier reported [46].

### 2.1. Instrumentation

Mass spectra using a Finnigan MAT 95 mass spectrometer, Waltham, MA, USA and electron ionisation (EI) method were registered over the temperature range of 303–623 K. The Cu content was determined with an iCE3300 FL atomic absorption spectrometer Thermo Fisher Scientific, Waltham, MA, USA. The content of C was found out with a Vario MACRO CHN ELEMENTAR Analysensysteme GmbH, Langenselbold, Germany. IR spectra were measured on a Vertex 70V spectrometer (Bruker Optik, Leipzig, Germany) using a reflective–single reflection diamond ATR unit (400–4000 cm^−1^). Thermal studies (TGA/DTA) were performed on an SDT 2960 TA analyser (New Castle, DE, USA) (dry N_2_; heating rate 2.5 K/min, heating range up to 598–692 K; sample mass 7–13 mg). Variable temperature infrared spectra (VT IR) were registered using a PerkinElmer Spectrum 2000 spectrometer (Waltham, MA, USA) over the range 400–4000 cm^−1^ with a medium slit width and a peak resolution of 2.0 cm^−1^. The glass vessel with the precursor sample (~100 mg) was placed in the homemade reactor tube and heated (from 303 to 753 K) under a dynamic vacuum (*p* = 10^−1^ mbar). IR spectra of vapours collected at selected temperatures were registered. Preliminary deposition experiments were carried out using the homemade horizontal hot-wall CVD reactor. Copper films were grown on Si(111) in argon within 60 min. The vaporisation temperatures T_V_ were 393 K and 453 K, whereas the deposition temperature values T_D_ were 593 K and 633 K. The morphology and composition studies of obtained materials were performed using scanning electron microscopy: SEM–LEO 1430VP, Ltd., Cambridge, UK (operating voltage 20 kV) equipped with an energy dispersive X-ray spectrometer (EDS) Quantax 200 with detector XFlash 4010 (Bruker AXS microanalysis GmbH, Berlin, Germany). XRD data were collected with a Philips X’PERT diffractometer with an X’Celerator Scientific detector (Malvern Panalytical Ltd., Malvern, UK). Diffractogramms were measured in the 10–100° 2θ range, using Cu K_α_ irradiation and sample spinning (step size—0.0167°, program. res. slit—0.5, measuring time—30 s per point). SEM images were registered using Quanta 3D FEG (FEI, Hillsboro, OR, USA). Transmission electron microscopy (TEM G2 F20X-Twin 200 kVFEI, Hillsboro, OR, USA) was used to determine the composition of the thermal analysis residue, confirm the composition of the deposited materials, and test the sensitivity of compounds to the high-energy electron beam. A sample for TEM testing was prepared by dissolving in anhydrous ethanol (99.8%), afterwards applying a drop on carbon-coated copper or nickel (CVD deposits) mesh with holes (Lacey type 400 mesh) and evaporating the solvent at room temperature. To identify the chemical elements, energy-dispersive X-ray spectroscopy (EDX, RTEM model SN9577, 134eV, EDAX, FEI, Hillsboro, OR, USA) spectra and selected area (electron) diffraction pattern were recorded. Atomic Force Microscopy (AFM) analysis of films was performed using a Veeco microscope (Veeco, Plainview, NY, USA).

### 2.2. DFT Calculations

The theoretical structures of complexes were optimised using density functional theory (DFT). All DFT calculations were performed using the Gaussian 16 (Wallingford, CT, USA) [47] available in PLGrid Infrastructure and the following sets of functionals and functional databases: B3LYP/6-31G, B3LYP/SDD(Cu) + 6-31G, RBW91/LANL2DZ, RBW91/LANL2DZ, MN15/6-31G, M06/6-31G, M06/6-311G(d,p), and M06/LANL2DZ. The lowest energy of the system was obtained when using the hybrid functional of Truhlar and Zhao M06 [48] and 6-311G(d,p) basis set. GaussView 5.0 (Wallingford, CT, USA) was used to visualise optimised structures.

### 2.3. Software

All graphical data were further processed with OriginPro 9.1 (Northampton, MA, USA). Diagrams of thermal and electron compounds decomposition were drawn using ChemDraw Ultra 12.0 (Mid-Cambridge, MA, USA). TA Universal Analysis (New Castle, DE, USA) was used to analyse thermograms. AFM images were prepared using the Gwyddion program (Brno, Czech Republic), while the roughness was determined using NanoScope Analysis (Bruker, Billerica, MA, USA). Grain size in the layers was determined by ImageJ (LOCI, University of Wisconsin, Madison, WI, USA). To assign the decomposition products in VT IR experiments, the registered spectra were compared with the spectra of molecules in the gas phase collected in SpectraBase [49].

### 2.4. Synthesis of [Cu_2_(NH_2_(NH=)CC_2_F_5_))_2_(µ-O_2_CR_F_)_4_]) (**1**–**4**)

Synthesis was carried out using Schlenk glassware and a glove box in an argon atmosphere. [Cu_2_(µ-O_2_CR)_4_] (0.5 mmol, R = CF_3_, C_2_F_5_, C_3_F_7_, C_4_F_9_) was placed in a round-bottomed flask, and 20 cm^3^ of anhydrous acetonitrile was added, followed by the introduction of pentafluoropropylamidine (AMDH, 1 mmol). The resulting dark-blue solution was stirred for 24 h. After this time, the solvent was removed under reduced pressure (1.3 × 10^−3^–1.3 × 10^−4^ mbar). The obtained compounds were green-blue solids, stable in air. Yields ranged between 66% and 83%. No crystals suitable for an X-ray structure determination were obtained.

**[Cu_2_(AMDH)_2_(µ-O_2_CCF_3_)_4_] (1)** Cu_2_O_8_N_4_H_6_C_14_F_22_ (calc./found) % Cu 14.1/15.1, C 18.6/18.3; EI MS T = 396 K (m/z, RI %) [AMDH]^+.^ (162, 36); [Cu(AMDH)]^+^ (225, 100); [Cu_2_(O_2_CCF_3_)]^+^ (239, 27); [Cu(AMDH)_2_]^+^ (387, 71); [Cu_2_(AMD)_2_]^+^ (448, 29); [Cu_2_(AMDH)_2_(O_2_CCF_3_)_3_]^+^ (789, 67); IR (3519, 3380, 3323, 3258, 3191, 3009, 1703, 1670, 1649, 1597, 1507, 1443, 1376, 1334, 1276, 1189, 1141, 1029, 974, 926, 846, 795, 772, 727, 686, 647, 637, 618, 599, 557, 522, 490, 429, 403 cm^−1^).

**[Cu_2_(AMDH)_2_(µ-O_2_CC_2_F_5_)_4_] (2)** Cu_2_O_8_N_4_H_6_C_18_F_30_ (calc./found) % Cu 11.5/11.5, C 19.6/18.4; EI MS T = 353 K (m/z, RI %) [AMDH]^+.^ (162, 39), [Cu(AMDH)]^+^ (225, 23), [Cu_2_(O_2_CC_2_F_5_)]^+^ (289, 5), [Cu(AMDH)_2_]^+^ (387, 14), [Cu_2_(AMD)_3_]^+^ (609, 7), [Cu_2_(AMDH)_2_(O_2_CC_2_F_5_)_3_]^+^ (939, 24); IR (3573, 3516, 3390, 3240, 3194, 2955, 1707, 1657, 1603, 1555, 1510, 1424, 1324, 1209, 1157, 1032, 974, 897, 822, 772, 737, 686, 650, 612, 589, 554, 544, 516, 432 cm^−1^).

**[Cu_2_(AMDH)_2_(µ-O_2_CC_3_F_7_)_4_] (3)** Cu_2_O_8_N_4_H_6_C_22_F_38_ (calc./found) % Cu 9.8/10.4, C 20.2/20.1; EI MS T = 383 K (m/z, RI %) [AMDH]^+.^ (162, 13), [Cu(AMDH)]^+^ (225, 4), [Cu_2_(O_2_CC_3_F_7_)]^+^ (339, <1), [Cu(AMDH)_2_]^+^ (387, 1), [Cu_2_(AMDH)(O_2_CC_3_F_7_)]^+^ (501, 1), [Cu_2_(AMDH)_2_(O_2_CC_3_F_7_)_3_]^+^ (1089, 2); IR (3516, 3387, 3317, 3252, 3198, 3059, 3012, 1710, 1675, 1606, 1504, 1411, 1337, 1270, 1209, 1183, 1166, 1119, 1083, 1029, 971, 936, 820, 772, 743, 721, 682, 650, 637, 602, 564, 532, 483, 429 cm^−1^).

**[Cu_2_(AMDH)_2_(µ-O_2_CC_4_F_9_)_4_] (4)** Cu_2_O_8_N_4_H_6_C_26_F_46_ (calc./found) % Cu 8.5/8.6, C 20.8/21.3; EI MS T = 396 K (m/z, RI %) [AMDH]^+.^ (162, 23), [Cu(AMDH)]^+^ (225, 54), [Cu(AMDH)_2_]^+^ (387, 25), [Cu_2_(O_2_CC_4_F_9_)]^+^ (389, 21), [Cu_2_(AMDH)(O_2_CC_4_F_9_)]^+^ (551, 9), [Cu(AMDH)_2_(O_2_CC_4_F_9_)]^+^ (650, 47), [Cu_2_(AMDH)_2_(O_2_CC_4_F_9_)_3_]^+^ (1239, 38); IR (3525, 3390, 3294, 3202, 2970, 1704, 1672, 1608, 1577, 1502, 1413, 1353, 1330, 1202, 1160, 1132, 1061, 1032, 974, 936, 913, 884, 874, 817, 772, 743, 714, 679, 650, 612, 554, 528, 426 cm^−1^).

## 3. Results and Discussion

In the acetonitrile environment, two amidine molecules are coordinated by dinuclear copper(II) carboxylate and complexes of formula [Cu_2_(AMDH)_2_(µ-O_2_CR)_4_], where R = CF_3_, C_2_F_5_, C_3_F_7_, C_4_F_9_, were formed:

$$[{\mathrm{Cu}}_{2}\left(\mu -O_{2}{\mathrm{CR}}_{F}{)}_{4}\right]+2\mathrm{AMDH}\to [{\mathrm{Cu}}_{2}{\left(\mathrm{AMDH}\right)}_{2}\left(\mu -O_{2}{\mathrm{CR}}_{F}{)}_{4}\right]$$

The obtained compounds (**1**)–(**4**) were blue-green air-stable solids.

### 3.1. Infrared Spectra Analysis

**Figure 1:**
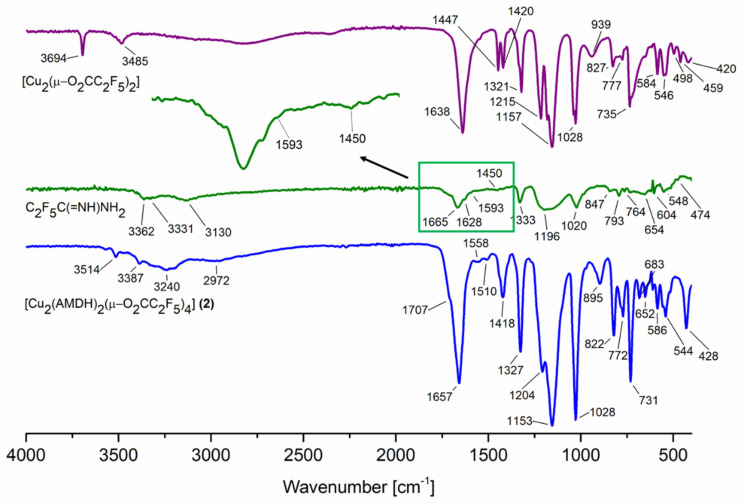
Infrared spectra for the reactants and the reaction product **[Cu_2_(AMDH)_2_(µ-O_2_CC_2_F_5_)_4_] (2)**.

In the infrared spectra (Supplementary Materials, Figures S1–S4), characteristic bands for asymmetric stretching vibrations of the carboxylate group were found over the range of 1649–1675 cm^−1^ (Table 1, Figure 1). Moreover, the value for symmetric stretching vibration was observed over the range of 1411–1443 cm^−1^. The calculated parameter Δν = ν_asCOO_ − ν_sCOO_, which is used for proposing of carboxylate coordination mode, was 206 cm^−1^ for the compound **[Cu_2_(AMDH)_2_(µ-O_2_CCF_3_)_4_] (1)**, which was close to the Δν_(RCOONa)_ value for sodium trifluoroacetate (223 cm^−1^). This suggests the bridging coordination mode of the carboxylate ligand. A similar relationship Δν ≅ Δν_(RCOONa)_ occurred for the next compounds (**2**)–(**4**) and the formation of the carboxylate bridges was proposed.

Bands characteristic of the amidine molecule were also observed in the spectrum: stretching vibration of the C=N bond (1703–1710 cm^−1^), stretching vibration of a =NH group (3009–3240 cm^−1^), deformation vibrations of the NH_2_ amino group (1597–1608 cm^−1^), and stretching of N=C–N group (1502–1510 cm^−1^). The coordination shifts of the signals mentioned above (relative to the free ligand) were in the range of 39–46 cm^−1^, 72–121 cm^−1^, 4–15 cm^−1^, and 52–60 cm^−1^, respectively (Table 1). The obtained results suggest the amidine coordination through the =NH group because the coordination shift for the vibrations of this group has the most significant value.

Interestingly, the band characteristic for the stretching vibrations of the =NH group shifts towards lower values for compounds (**1**) and (**3**) with an odd number of carbon atoms in the carboxylate chain and higher values for compounds (**2**) and (**4**) with an even number of carbon atoms in the carboxylate chain. There is also a difference in the area of the stretching bonds of the =NH and NH_2_ groups (Figure 1 and Supplementary Materials, Figures S1–S4). It seems that, for compounds (**1**) and (**3**), the amidine arrangement in the structure is different than for compounds (**2**) and (**4**).

### 3.2. DFT Calculations

**Figure 2:**
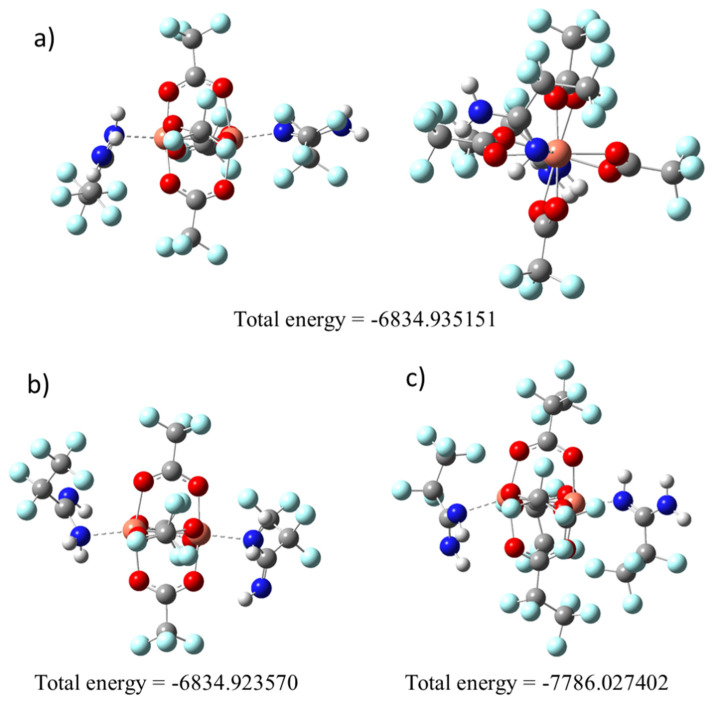
Optimised compounds structures by the density functional theory (DFT) M06/6-311G(d,p) method: (**a**) **[Cu_2_(AMDH)_2_(µ–O_2_CCF_3_)_4_] (1)**, =NH bonded; (**b**) **[Cu_2_(AMDH)_2_(µ–O_2_CCF_3_)_4_] (1)**, –NH_2_ bonded; (**c**) **[Cu_2_(AMDH)_2_(µ–O_2_CC_2_F_5_)_4_] (2)**, =NH bonded.

To confirm the way of amidine coordination, we decided to optimise the geometry using computational chemistry methods. For the complex **[Cu_2_(AMDH)_2_(µ–O_2_CCF_3_)_4_] (1)**, calculations were started for the two structures in which the amidine coordination occurred through the =NH group (Figure 2a) or –NH_2_ (Figure 2b). The calculations showed that the lower energy structure is that in which the amidine was bound via the = NH group (Figure 2). Therefore, in the case of complex (**2**), calculations were made only for the molecule with an amidine coordinated by the =NH bond (Figure 2c) owing to the size of the molecule, and thus the long computation time.

It should be noted that the calculated structures are asymmetric, there is a distortion of the coordinated carboxylate, and the Cu-O bonds are not equal (Figure 2). There are also differences in the spatial arrangement of amidines. The optimised structures confirm the different arrangement of the amidines in the structure for the compound (**1**) and (**2**). One of the amidine groups H_2_N–C=NH is perpendicular to the copper atoms, while the other is parallel.

**Figure 3:**
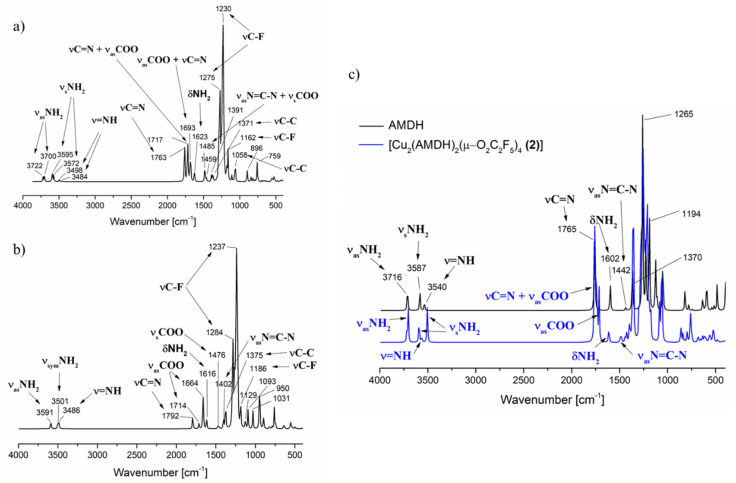
Theoretical infrared spectrum calculated by DFT M06/6-311G(d,p): (**a**) **[Cu_2_(AMDH)_2_(µ-O_2_CCF_3_)_4_] (1)**, =NH bonded amidine, (**b**) **[Cu_2_(AMDH)_2_(µ-O_2_CCF_3_)_4_] (1)**, –NH_2_ bonded amidine; (**c**) **[Cu_2_(AMDH)_2_(µ-O_2_CC_2_F_5_)_4_] (1)**, =NH bonded (blue line) and AMDH (black line).

Infrared spectra (Figure 3) were also calculated for the two alternative optimised structures to confirm the assigned bands in the experimental spectra of the complexes (Table 1). In the area of stretching bonds characteristic of the –NH_2_ and =NH groups for compound (**1**), in the case of amidine coordination by the =NH group (Figure 2a), a slight shift only in the position of the asymmetric stretching band of the –NH_2_ group vibrations in relation to the free ligand is calculated (3716 cm^−1^, AMDH; 3722 cm^−1^, 3700 cm^−1^, complex (**1**)). On the other hand, the band characteristic for the stretching vibrations of the =NH group shifts towards lower values (3540 cm^−1^ → 3498 cm^−1^, 3485 cm^−1^). In the case of compound (**1**) alternative structure (Figure 2b) in which the amidine was coordinated by the –NH_2_ group, a significant shift of the NH_2_ asymmetric stretching vibrations towards lower values was observed (3716 cm^−1^ → 3591 cm^−1^). Comparing the theoretical and experimental infrared spectra for complex (**1**), it was confirmed that the amidine coordination occurs through the =NH group, because, in the experimental spectra, no significant shift of the band characteristic for the –NH_2_ group was detected (Table 1).

Interestingly, in the theoretical spectra, a difference was also observed in the shift of the stretching =NH band for complex (**1**) and (**2**) as in the experimental spectra. In compound (**2**), this band shifts towards higher values (3540 cm^−1^ → 3563 cm^−1^). Moreover, in the theoretical spectrum for complex (**1**), there is a double set of bands for asymmetric and symmetric stretching vibrations of the NH_2_ group. However, in the case of (**2**), only for –NH_2_ symmetrical stretching vibrations, a double set of bands was observed. As a result, more bands in the region >3000 cm^−1^ were observed in the spectrum for complex (**1**) than for complex (**2**), and it was also visible in the experimental spectra.

In the case of the stretching vibrations characteristic for the C=N group, no coordination shift was observed in the theoretical spectra (Figure 3). It should be noted that the νC=N bands overlap with the asymmetric stretching vibrations of the carboxylic groups. Because the theoretical spectra were calculated for molecules in the gas phase, the individual bands are better separated. In the solid-phase spectrum (Figure 1), one band with the shoulder was observed, making it difficult to distinguish individual vibrations.

The theoretical spectra also confirmed the proposed assignment of bands characteristic for deformation vibrations of the –NH_2_ group and stretching vibrations of the N=C–N group (Section 3.1). The ν_as_N=C–N shifts more towards higher values (1442 cm^−1^ → 1485 cm^−1^) than the δNH_2_ (1602 cm^−1^ → 1623 cm^−1^).

In conclusion, both the calculated energies and the band coordination shifts in the experimental and theoretical spectrum confirmed the coordination of the amidine by the =NH group.

### 3.3. Thermal Analysis

**Figure 4:**
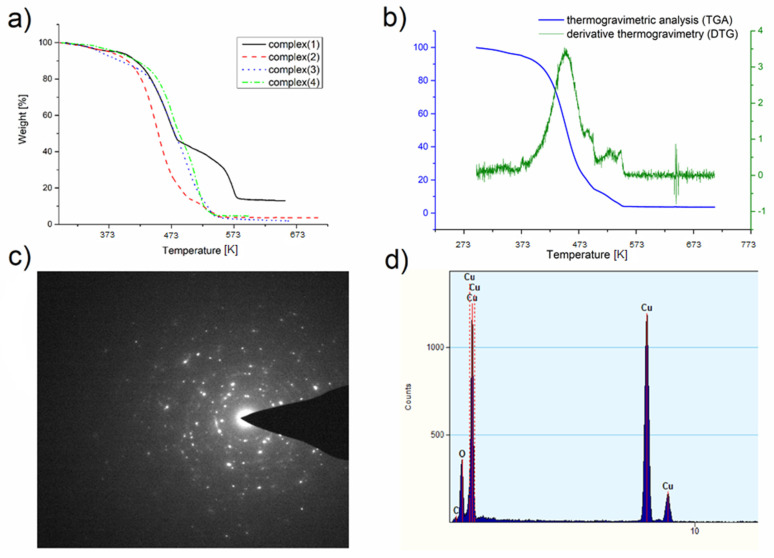
(**a**) Thermogravimetric analysis (TGA) for the complexes: (**1**), (**2**), (**3**), (**4**); (**b**) derivative thermogravimetry (DTG) and TGA for the complex (**2**) showing the complexity of the decomposition process; (**c**) transmission electron microscope (TEM) diffraction pattern for the residue from TGA of (**2**); (**d**) energy-dispersive X-ray spectroscopy (EDX) spectrum for the residue after TGA of (**2**).

Thermal analysis was performed to examine the behavior of the complexes during heating under a nitrogen atmosphere and to determine the final decomposition temperature and the degradation product. Only for complex (**1**), two decomposition stages can be distinguished (Figure 4a). For the other compounds ((**2**)–(**4**)), the thermal decomposition consists of several overlapping steps particularly visible in the derivative graph (Figure 4b). This may be because, for complexes (**2**), (**3**), and (**4**), a small amount of residue was observed (Table 2) after the thermal analysis, which means that the complex can be partially evaporated under atmospheric pressure. However, under these conditions, in addition to evaporation, compound decomposition also occurs.

To confirm the composition of the final product of thermal analysis, transmission electron microscope (TEM) was used owing to the small amount left in the crucible. TEM diffraction pattern and EDX spectra (Figure 4c,d) revealed that the decomposition product under the conditions of thermal analysis (atmospheric pressure, nitrogen atmosphere) is copper(I) oxide.

The temperature of the decomposition process onset changed over the range of 337–391 K, and the temperature of the final product formation varied from 551 K (**3**) to 586 K (**1**) (Table 2). For comparison, in the case of complexes mentioned in the Introduction with amines [Cu_2_(^t^BuNH_2_)_2_(µ-O_2_CR_F_)_4_], these values were 318–396 K and 458–668 K, respectively. Interestingly, in the case of complexes with CF_3_ and C_2_F_5_ substituents, lower T_i_ and T_f_ temperatures were observed for the complexes with tert-butylamine in the axial position. However, for the C_3_F_7_ and C_4_F_9_ chains, lower temperatures were registered for the complexes with amidine [41].

### 3.4. Mass Spectra Analysis

Mass spectra (EI-MS) of the studied compounds were registered between 303 and 623 K and applied to determine the composition of the complex and identify metallated fragments. The benefit of this approach is that the compound is first evaporated and then exposed to electrons (70 eV).

**Figure 5:**
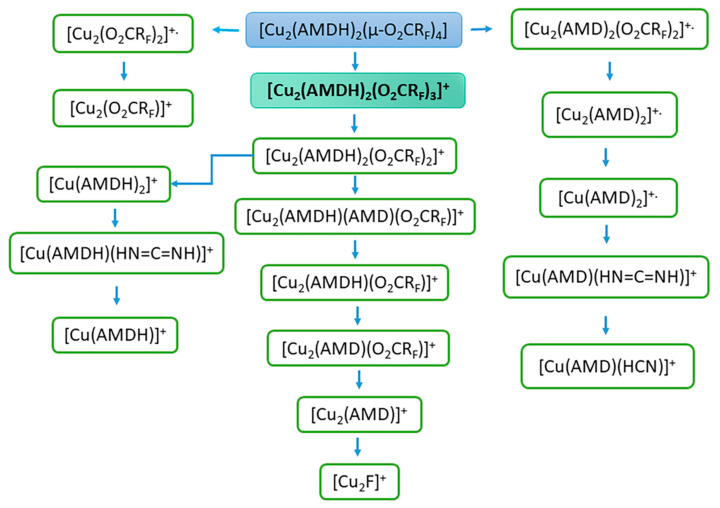
Fragmentation scheme for metallated fragments of [Cu_2_(AMDH)_2_(µ-O_2_CR_F_)_4_].

For all compounds, the pseudomolecular ion of the [Cu_2_(AMDH)_2_(O_2_CR_F_)_3_]^+^ formula, where R_F_ = C_n_F_2n+1_, *n* = 1–4, formed by the detachment of one carboxylate ligand, was visible in EI MS spectra (Table 3 and Tables S1–S3). Pseudomolecular ions of this type were previously observed for [Cu_2_(^t^BuNH_2_)_2_(µ-O_2_CR_F_)_4_] complexes with tert-butylamine in the axial position [41]. For compounds (**1**) and (**2**), this ion is already present at low temperatures: 313 K (RI = 4%) and 327 K (RI = 8%), respectively. The [Cu_2_(AMDH)_2_(O_2_CR_F_)_3_]^+^ ion reaches its highest intensity over the temperature range of 353–396 K with only 2% RI for compound (**3**) and 67% RI for compound (**1**). The low intensity of the pseudomolecular ion for compound (**3**) may result from its high sensitivity to the electron beam, which caused its rapid decomposition. This is also evidenced by the high intensity of the [Cu_2_F]^+^ ion, which is the end product of the decomposition (Figure 5) and for the compound (**3**) reached 97% RI at 550 K (Table S2). Generally, there are also other detected dinuclear and mononuclear metallated fragments containing both ligands, such as [Cu_2_(AMDH)_2_(O_2_CR_F_)_2_]^+^, [Cu_2_(AMDH)(O_2_CR_F_)_3_]^+^, [Cu(AMDH)_2_(O_2_CR_F_)]^+^, and [Cu(AMDH)(O_2_CR_F_)]^+^. This fact confirms the coordination of the entire amidine molecule to the perfluorinated copper(II) carboxylate. Moreover, the intact molecule of the tested compound passes into the gas phase. Interestingly, ions containing Cu(I) detection, such as [Cu_2_(AMDH)_2_(O_2_CR_F_)]^+^ and [Cu_2_(AMDH)(O_2_CR_F_)]^+^, suggest Cu(II) reduction during the thermolysis of the compounds. In the case of compounds (**3**) and (**4**), even trinuclear and tetranuclear ions [Cu_3_(AMDH)(O_2_CC_4_F_9_)_5_]^+^ (RI_max_ = 3, 428 K), [Cu_3_(AMDH)(O_2_CR_F_)_4_]^+^ (RI_max_ = 17, 460 K), and [Cu_4_(NH_2_)_2_(AMDH)(AMD)_2_(O_2_CC_4_F_9_)_2_]^+^ (RI_max_ = 4, 460 K) were observed. This may indicate the polymeric structure of these complexes.

Ions containing deprotonated amidine (i.e., amidinate) having the following composition: [Cu_2_(AMD)(O_2_CR_F_)_3_]^+.^, [Cu_2_(AMD)_2_(O_2_CR_F_)_2_]^+.^, [Cu_2_(AMD)_2_(O_2_CR_F_)-H]^+^, [Cu_2_(AMD)(O_2_CR_F_)_2_-H]^+^, and [Cu_2_(I,II)(AMD)(O_2_CR_F_)]^+^/[Cu_2_(I)(AMD)(O_2_CR_F_)]^+.^ (the highest intensities they achieved in the temperature range of 398–486 K) were detected in the spectra. The obtained results exhibited the deprotonation of the coordinated amidine ligand during heating (vide infra, Section 3.5) under measurement conditions.

It is also worth noting the formation of ions containing coordinated ligands that were formed as a result of amidine decomposition, for example, [Cu_2_(NH_2_)(AMD)_2_(O_2_CCF_3_)]^+^, [Cu(AMDH)(HN=C=NH)]^+^, and [Cu(AMD)(HCN)]^+^. This may indicate that the formed Cu-N bond is strong. Therefore, the bonds in the ligand molecule were broken, and the amidine/amidinate was not completely detached.

Interestingly, at low temperatures (313–371 K), copper(I) and copper(II) fragments containing only N-donor ligands are also formed, such as [Cu_2_(AMD)_3_]^+^ [Cu_2_(AMD)_2_]^+.^, [Cu(AMDH)_2_]^+^, [Cu(AMD)_2_]^+.^, and [Cu(AMDH)]^+^. Moreover, in the case of compound (**1**), the [Cu(AMDH)]^+.^ fragment reaches 100% RI at 393 K, while for compound (**4**), it achieves 99% RI at 428 K. On the other hand, ions containing only the carboxylate ligand reach their highest intensity at higher temperatures. In the case of [Cu_2_(O_2_CR_F_)_2_]^+.^ ion, the relative intensity varies from 18 to 96% in the temperature range of 505–550 K (Table 3 and Tables S1–S3). In turn, the ion [Cu_2_(O_2_CR_F_)]^+^ reaches the highest intensity (50–75% RI) over the 428–538 K temperature range. The obtained results may indicate that the metal species with the amidinate ligand are more volatile than those with the carboxylate.

At the beginning of heating, fragments such as [AMDH]^+^, [C_2_F_5_]^+^, [CO_2_]^+.^, [CO_2_H]^+^, [HN=C=NH]^+.^, and [F_2_]^+.^ appear, which indicate the degradation of the compounds. However, it is difficult to distinguish which ions result from thermal decomposition and which are the result of interactions with electrons. The appearance of carbodiimide [HN=C=NH]^+^ suggests carbodiimide detachment as one of the mechanisms of degradation [50]. However, there is also release of amidine as a decomposition factor. The presence of [CO_2_H]^+^ is evidence of amidine deprotonation by carboxylate and the formation of carboxylic acid and amidinate.

[Cu_2_F]^+^ fragment was also detected in MS EI spectra for all studied compounds. It reaches an intensity of 13 to 97% RI at high temperatures (505–550 K) (Table 3 and Tables S1–S3). In the case of compound (**2**), [Cu]^+^ ion was also observed. The formation of this type of ions indicates that electrons can cause the decomposition of compounds into copper(I) fluoride or copper (vide infra). This is a desirable property for the potential precursors used in the FEBID method.

### 3.5. Variable Temperature Infrared Spectroscopy (VT IR)

To examine the composition of the gas phase formed during the heating of the complexes, variable temperature infrared spectroscopy was carried out. The measurement conditions are similar to the deposition parameters during the CVD experiments.

**Figure 6:**
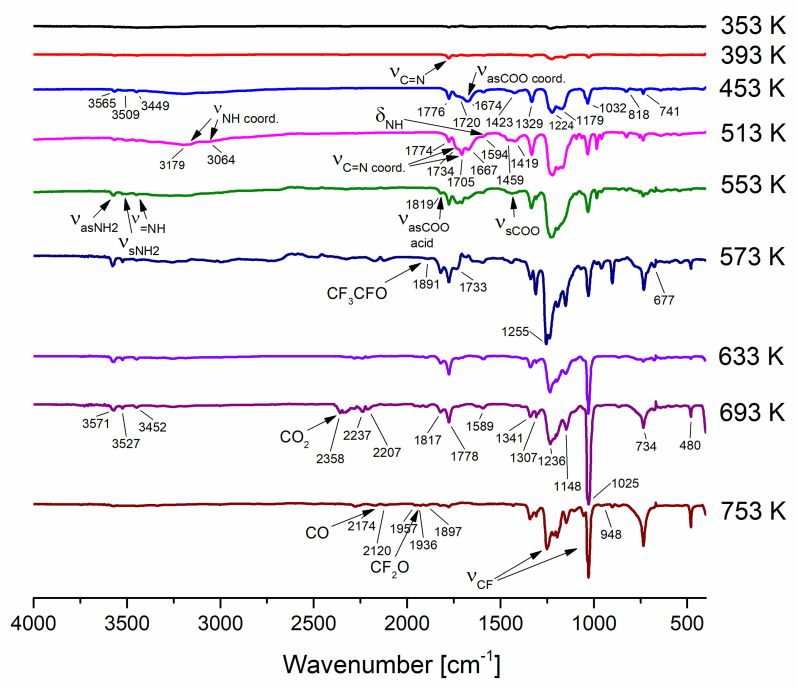
VT IR spectra of **[Cu_2_(AMDH)_2_(µ-O_2_CC_2_F_5_)_4_]** (**2**) in the temperature range of 353–753 K.

In the first step of decomposition (***stage I***), the band characteristics of the amidine molecule (3565 cm^−1^; ν_as_NH_2_, 3527 cm^−1^; ν_s_NH_2_, 3446 cm^−1^; ν=NH, 1776 cm^−1^; νCN, 1590 cm^−1^, δNH) were observed for compounds (**1**), (**3**), and (**4**) in the temperature range of 333–413 K (Figures S1–S3). In the case of compound (**2**), in addition to the bands mentioned above, there are the following observed signals: 3191 cm^−1^ (νNH_2_), 1734 cm^−1^ (νCN + ν_as_COO), and 1683 cm^−1^ (ν_as_COO) characteristic for complex **(2),** and signals 3515 cm^−1^ (νNH_2_) and 1705 cm^−1^ (νCN) assigned to the coordinated amidinate (Cu–AMD) species. It is difficult to propose a mode of amidinate coordination owing to the lack of spectroscopic data in the literature for copper compounds with perfluorinated AMD. These species occur in the gas phase over the temperature range of 353–533 K (Figure 6 and Figure S8).

Similarly, the second decomposition step (***stage II***) is comparable for compounds (**1**), (**3**), and (**4**). HAMD’s characteristic bands are still being observed. The entire molecules of complexes (**1**), (**3**), and (**4**) are also present at this stage in the temperature range of 433–453 K (**1**), (**3**), and 433–473 K (**4**). Moreover, in the gas phase, Cu–AMD species occur (Figures S5–S7). In the case of complex (**2**), in addition to the molecules mentioned above, signals, 1824 cm^−1^ (ν_as_COO) and 1440 cm^−1^ (ν_s_COO) bands were observed in the gas phase at 553 K, characteristic for carboxylic acid (Figure 6) [51].

At higher temperatures, there are more differences in the way compounds are decomposed. Generally, compounds (**1**), (**3**), and (**4**) have more decomposition stages than complex (**2**).

For compound (**2**) in the temperature range of 573–653 K (***stage III***), there are characteristic bands for Cu–AMD species, Cu_2_(O_2_CR_F_)_4_ (1651 cm^−1^; ν_as_COO) [42], HAMD, R_F_COOH [51], CF_3_CFO (1889 cm^−1^, 736 cm^−1^, 676 cm^−1^) [52], CF_2_O (1957 cm^−1^, 1936 cm^−1^) [52], CO_2_ (2322 cm^−1^) [53], and CO (2174 cm^−1^, 2126 cm^−1^) [54]. In turn, in the 673–753 K temperature range (***stage IV***), there are no more metalled species. Only decomposition products mentioned above such as HAMD, R_F_COOH, CF_3_CFO, CF_2_O, CO_2_, and CO occurred (Figure 6).

In the case of compound (**1**) in the third step of decomposition (***stage III***), there are characteristic bands for the entire complex (**1**) molecules, Cu–AMD, HAMD, and the carboxylic acid, at the temperature range of 473–533 K. The fourth stage of decomposition (***stage IV***) occurs in the temperature range of 553–593 K. The gas-phase contained the following species: Cu–AMD, Cu_2_(O_2_CCF_3_)_4_, HAMD, CF_3_COOH [55], CF_3_CFO, CO_2_, and CO. The next stage (***stage V***, 613–633 K) differs from the previous one by the lack of metal carriers. In the last tested temperature range (***stage VI***, 653–753 K) in the gas phase, HAMD, CF_3_COOH, CO_2_, and CO (Figure S5) were detected.

Compared with compound (**1**), in the third stage of complex (**3**) decomposition (***stage III***, 473–493 K), an additional band (2272 cm^−1^) was observed in the VT IR spectra. This band is characteristic of a gaseous C_2_F_5_CN. [56] At 513 K (***stage IV***), the gas phase consists of the following species: complex (**3**), Cu–AMD, HAMD, C_3_F_7_COOH [57], C_2_F_5_CN, and CO_2_. In turn, in the temperature range of 533–553 K (***stage V***), in addition to the aforementioned degradation products, CO molecule was detected. In the next step of decomposition (***stage VI***, 573–593 K), copper(II) perfluorobutyrate (1658 cm^−1^) was the only metal carrier. Additionally, the bands 1795 cm^−1^ (C=C stretching vibrations) and 1396 cm^−1^ (asymmetric C-F stretching mode in the CF_3_ group) were recorded in the VT IR spectra characteristic of the CF_2_=CF–CF_3_ molecule. [58] R_F_COOH, CF_3_CFO, C_2_F_5_CN, CO_2_, and CO were also observed in the gas phase at these temperatures. With increasing temperature (613–653 K, ***stage VI***), the composition of the gas phase changes slightly compared with ***stage V***, and there are no CF_3_CFO molecules (Figure S6).

In the case of compound (**4**), the molecules Cu–AMD, HAMD, and C_4_F_9_COOH occur in the third phase of degradation (***stage III***, 493–533 K). In the next stage of decomposition (***stage IV***, 553–573 K), the gas phase is composed of Cu–AMD, HAMD, C_4_F_9_COOH [59], CF_3_CFO, C_2_F_5_CN, CO_2_, and CO. At higher temperatures (***stage V***, 593–613 K), the amount of gaseous products decreased, and Cu–AMD, HAMD, carboxylic acid, and CF_3_CFO were observed. Finally, in the last stage of decomposition (***stage VI***, 633–753 K), the gas phase consists only of CF_2_=CF–CF_3_ and C_4_F_9_COOH (Figure S7).

**Figure 7:**
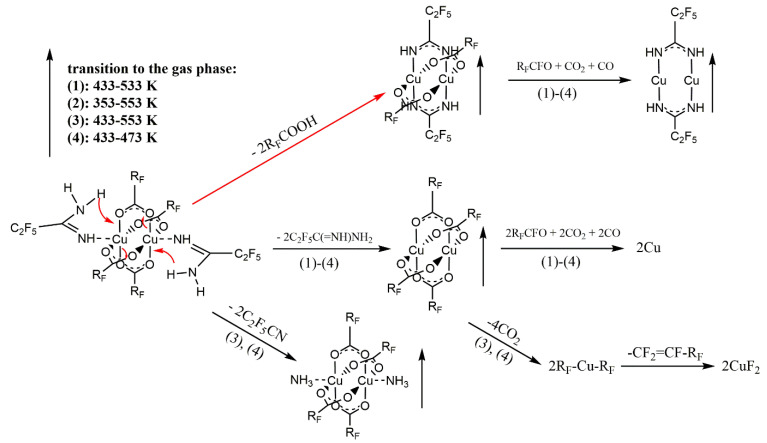
Proposed mechanism of [Cu_2_(AMDH)_2_(µ-O_2_CR_F_)_4_] thermal decomposition based on VT IR and EI MS studies.

Based on the VT IR spectra and the ions observed in the gas phase during the EI MS experiments (Section 3.4), the thermal decomposition mechanism of compounds **1**–**4** was proposed, as shown in Figure 7.

**Figure 8:**
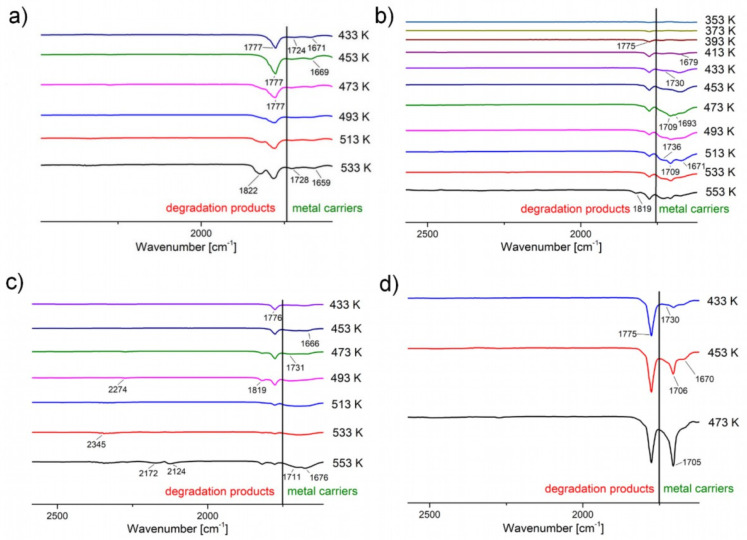
Enlargement of the area of occurrence of metal carriers (**green**) and degradation products’ (**red**) characteristic bands: (**a**) **[Cu_2_(AMDH)_2_(µ-O_2_CCF_3_)_4_] (1)**, (**b**) **[Cu_2_(AMDH)_2_(µ-O_2_CC_2_F_5_)_4_] (2)**, (**c**) **[Cu_2_(AMDH)_2_(µ-O_2_CC_3_F_7_)_4_] (3)**, (**d**) **[Cu_2_(AMDH)_2_(µ-O_2_CC_4_F_9_)_4_] (4)**.

Based on the results described above, it can be concluded that complex (**2**) has the highest volatility. It was observed in the gas phase in the broadest temperature range. Moreover, the bands characteristic of this compound have a higher intensity than the bands assigned to degradation products in the temperature range of 433–533 K (Figure 8b). The inverse relationship was observed for compounds (**1**), (**3**), and (**4**) (Figure 8a,c,d). This means that complex (**2**) demonstrates the highest concentration in the gas phase. It is also worth adding that the concentration of other metal carriers is equally high. For this reason, this compound was selected as the best of the newly synthesised potential CVD precursors. Another compound ensuring a satisfactory amount of metal carriers in the gas phase was complex (**3**), which was also selected for testing in the CVD process.

The obtained complexes (**1**–**4**) have higher volatility than analogues with tert-butylamine, because the complexes [Cu_2_(AMDH)_2_(µ-O_2_CR_F_)_4_] go into the gas phase in the temperature range of 353–553 K, while the [Cu_2_(NH_2_^t^Bu)_2_(µ-O_2_CR_F_)_4_] compounds are a source of metal carriers over the temperature range of 373–553 K [42].

It is also worth noting that relatively small changes in the compound structure (the carbon chain length increased in the carboxylate) affect the mechanism of compound decomposition. One interesting observation is the lack of a simple correlation between the carbon chain length in the carboxylate and complex volatility. Both complex (**1**) and complex (**4**) have a low concentration in the gas phase.

Compared with the mass spectrometry results, no characteristic signals for carbodiimide or perfluorinated hydrocarbons (for compounds (**1**) and (**2**)) were detected in the VT IR spectra. It can be concluded that these products are formed only as a result of electron-induced decomposition rather than thermal decomposition. This observation confirms that VT IR spectra are an effective method for testing CVD precursors because the measurement conditions (10^−1^ mbar) are close to those in the used CVD reactor.

### 3.6. Sublimation Experiments

**Figure 9:**
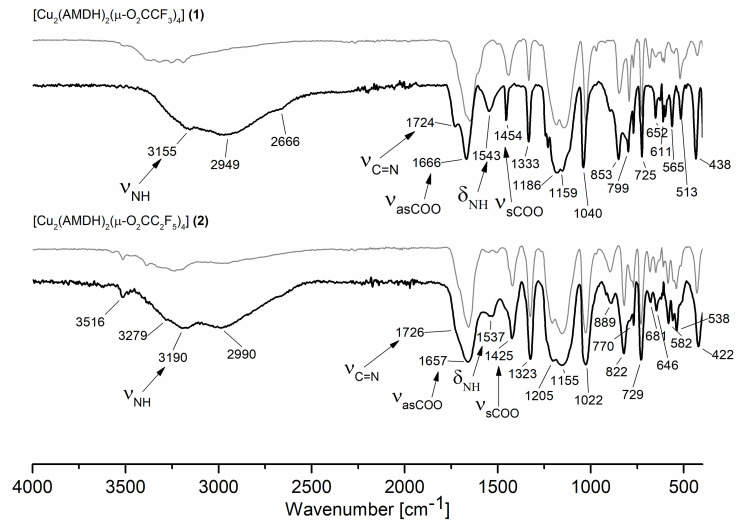
Infrared spectra for the compound **[Cu_2_(AMDH)_2_(µ-O_2_CCF_3_)_4_] (1)** (**top**) and **[Cu_2_(AMDH)_2_(µ-O_2_CC_2_F_5_)_4_] (2)** (**bottom**) before sublimation (**gray**) and after sublimation (**black**).

To further investigate the volatile species, sublimation experiments at 393 K and 1.3 × 10^−4^ mbar were performed for compounds (**1**) and (**2**). Then, IR spectra were taken for the compounds deposited on the cooling finger. As can be seen for compound (**2**), the spectra before and after sublimation are similar (Figure 9 bottom). There are only slight differences in the positions and intensities of the bands assigned to the N-H stretching and deformation vibration. Therefore, it can be concluded that compound (**2**) evaporates without decomposition. In the case of compound (**1**), the changes in the IR spectra before and after sublimation are much more noticeable (Figure 9 top). It is worth mentioning that there are bands in the spectrum of the sublimation product characteristic for both coordinated ligands (3155 cm^−1^, ν_N-H_; 1724 cm^−1^, ν_C=N_; 1666 cm^−1^, ν_C=O_; 1543 cm^−1^, δ_NH_). This proves that compound (**1**) is a source of the metal carrier in the gas phase. It is likely that, during heating, a rearrangement in the structure of the compound occurs. The reason may be the breaking of hydrogen bonds between the molecules of the heated compound [60].

### 3.7. TEM and SEM Observations

To initially assess the sensitivity of compound (**2**) to the high-energy electron beam, observations were made using a scanning electron microscope (SEM, 20 keV) and a transmission electron microscope (TEM, 200 keV). Complex (**2**) was selected for testing because it demonstrates the best volatility. For SEM analysis, the compound was prepared by depositing its layer on a silicon wafer using the earlier described sublimation method (Section 3.6). For TEM analysis, the compound was dissolved in ethanol, and the resulting solution was applied to a mesh and allowed to solvent evaporate.

**Figure 10:**
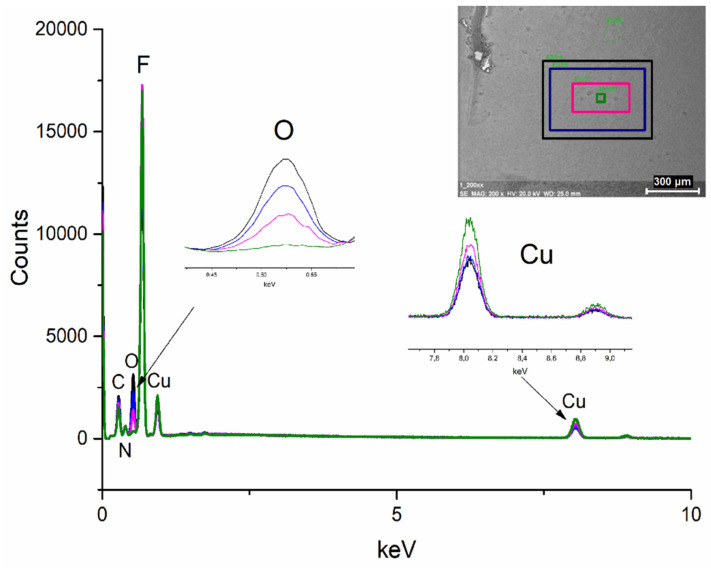
Examined scan areas’ EDX spectra for **[Cu_2_(AMDH)_2_(O_2_CC_2_F_5_)_4_] (2)** deposited on a silicon wafer.

EDX spectra were taken during the SEM analysis, each of them recorded for 30 s. During the experiment, the scanning area was reduced, which increases the dose of electrons (Figure 10). With the increasing dose of electrons, the oxygen content decreases, the carbon content is slightly reduced, and the copper content increases. The contents of the other elements were almost the same.

**Figure 11:**
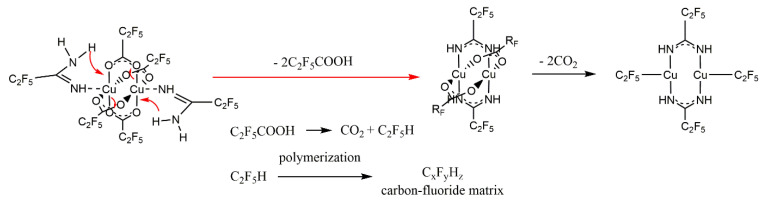
Proposed mechanism of **[Cu_2_(AMDH)_2_(O_2_CC_2_F_5_)_4_] (2)** decomposition under the influence of an electron beam in the SEM microscope.

Based on the obtained results and the mass spectra (Section 3.4), the following mechanism of the compound decomposition under SEM conditions was proposed. In the first stage, a proton transfer from the amidine molecule to the carboxylate probably took place, resulting in the formation of a coordinated deprotonated amidine and C_2_F_5_COOH (Figure 11). Ions with deprotonated amidine were observed in EI MS spectra (Table 3). Acid was decomposed by electrons, forming CO_2_ and C_2_F_5_H. This is confirmed by the EI MS spectra of the complexes (Section 3.4) and C_2_F_5_COOH [61], in which no molecular ion derived from the acid was observed. The unchanged amount of fluorine before compound interaction with electrons may indicate that C_2_F_5_H has not been released from the layer as a gas. Stoffels et al. proved by electron attachment mass spectrometry experiment that, in radio frequency fluorocarbon plasmas of CF_4_, C_2_F_6_, and C_4_F_8_, polymerisation occurred readily, and molecules containing up to ten carbon atoms have been found [62]. Therefore, it is possible that, during the interaction of C_2_F_5_H with the electron beam, polymerisation has occurred, and a carbon-fluorine matrix was formed.

In the second stage of decomposition, decarboxylation occurred, and the perfluorinated groups were still bound to the central atom. Formation of [Cu(CF_3_)_2_]^−^ ion, with fluorocarbon group bonded to the copper atom, has been proved by Rijs et al. in their research on the mechanism of decomposition of [Cu_2_(µ-O_2_CCF_3_)_4_] using mass spectrometry [63]. Moreover, in the electron collision experiments with [Cu_2_(µ-O_2_CC_2_F_5_)_4_], the [Cu(CF_3_)_2_]^−^ ion was formed [64].

It is seen that, during the interaction of the electron beam with a solid-phase compound, the amount of energy under SEM conditions is insufficient to remove the coordinated ligands effectively. The obtained results showed that the Cu-O bond is the weakest in the complex. The proposed mechanism of compound decomposition under the influence of the SEM electron beam is presented in Figure 11. It still seems necessary to conduct FEBID experiments because, in this method, the precursor is deposited from the gas phase and, as evidenced by the EI MS spectrum (Section 3.4), in which the precursor is in gaseous form and interact with low-energy electrons, it is possible to decompose it into copper(I) fluoride.

**Figure 12:**
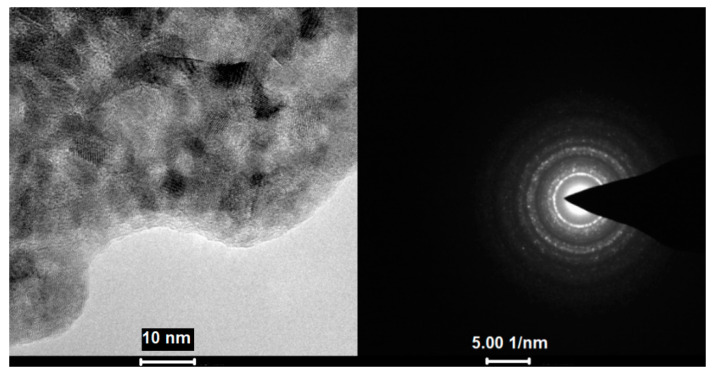
Image of the sample after a few seconds of **[Cu_2_(AMDH)_2_(O_2_CC_2_F_5_)_4_] (2)** interaction with electrons and diffraction pattern, which confirms the crystallinity of the deposit.

During TEM imaging, structural changes of the sample were visible from the beginning of the measurement. After a few seconds, only the image of the sample was stabilised, and crystallites were observed (Figure 12). The obtained diffraction confirmed the crystallinity of the formed substance with diffraction rings matching the cubic CuF_2_.

**Figure 13:**
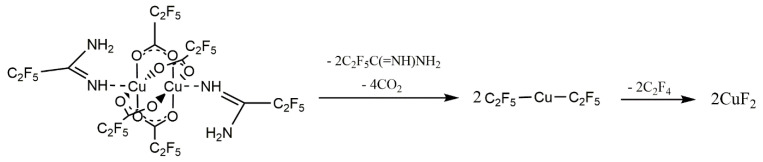
Proposed mechanism of **[Cu_2_(AMDH)_2_(O_2_CC_2_F_5_)_4_] (2)** decomposition under the influence of an electron beam under the TEM analysis conditions.

The EDX spectra of the TEM obtained material showed the following atomic contents of the elements: 11.6 at.% Cu, 41.2 at.% F, and 47.2 at.% C. Such values suggest that, apart from copper(II) fluoride formation, after the compound (**2**) interaction with the high-energy electron beam, a matrix consisting of fluorine and carbon is formed. However, it should be taken into account that the TEM grid was covered with carbon, which affects the semi-quantitative measurement. It is worth noting that the resulting material does not contain oxygen and nitrogen, which were in the pristine compound (**2**). This may indicate the breaking of Cu-N and Cu-O bonds under the conditions of TEM observation. The proposed mechanism of compound decomposition under the influence of the TEM electron beam is presented in Figure 13.

The microscopic observations showed that compound (**2**) is sensitive to the high-energy electron beam. It is visible that the decomposition of the compound strongly depends on the beam power and electron dose. When high electron energy (TEM) is used, CuF_2_ is the final decomposition product, but in SEM, CO_2_ detachment and organometallic species formation were suggested, while electrons have low energy (EI MS, Section 3.4), and Cu_2_F_2_ is formed.

### 3.8. CVD Preliminary Experiments

Based on the volatility studies (TA, EI MS, VT IR–Section 3.3, Section 3.4 and Section 3.5), complexes (**2**) and (**3**) were selected for the hot-wall CVD experiments (Table 4). In the case of complex (**2**), the evaporation temperature of 393 K was sufficient to deposit the layer, while for precursor **(3)_,_** it was necessary to increase the temperature to 453 K. This is consistent with the results of the VT IR analysis (Section 3.5), because for complex (**2**), the bands characteristic for the copper carriers appeared at lower temperatures (353 K) than for the complex (**3**) (433 K), and additionally they had higher intensities, which indicates a higher concentration of these metal carriers in the gas phase. The process was carried out using decomposition temperatures from 573 K to 633 K. The best results were obtained using 633 K as the deposition temperature T_D_.

**Figure 14:**
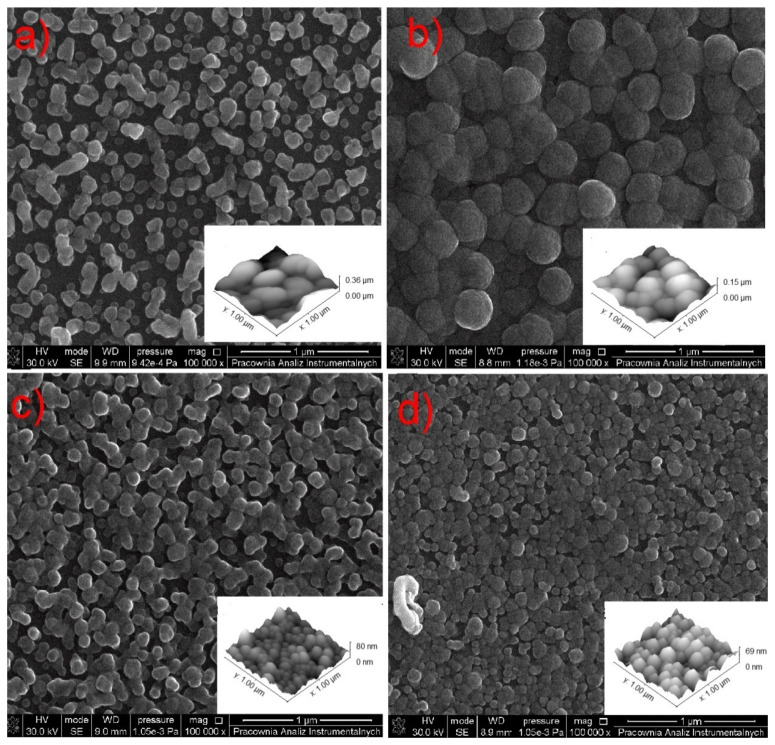
SEM and AFM results of preliminary chemical vapour deposition (CVD) experiment using **[Cu_2_(AMDH)_2_(O_2_CC_2_F_5_)_4_] (2)**: (**a**) T_V_ = 453 K, T_D_ = 593 K; (**b**–**d**) T_V_ = 393 K, T_D_ = 633 K, in the case of (**b**), the transport way of vapours was shorter.

**Figure 15:**
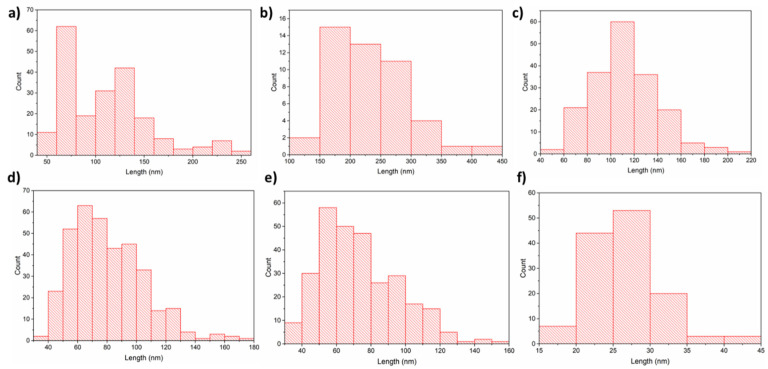
Histograms of a grain-size distribution: (**a**) **[Cu_2_(AMDH)_2_(O_2_CC_2_F_5_)_4_] (2)**, T_V_ = 453 K, T_D_ = 593 K; (**b**–**d**) **[Cu_2_(AMDH)_2_(O_2_CC_2_F_5_)_4_] (2),** T_V_ = 393 K, T_D_ = 633 K, in the case of (b), the transport way of vapours was shorter; (**e**) **[Cu_2_(AMDH)_2_(µ-O_2_CC_3_F_7_)_4_] (3)**, T_V_ = 453 K, T_D_ = 633 K; (**f**) **[Cu_2_(AMDH)_2_(µ-O_2_CC_3_F_7_)_4_] (3)** T_V_ = 393 K, T_D_ = 573 K.

**Figure 16:**
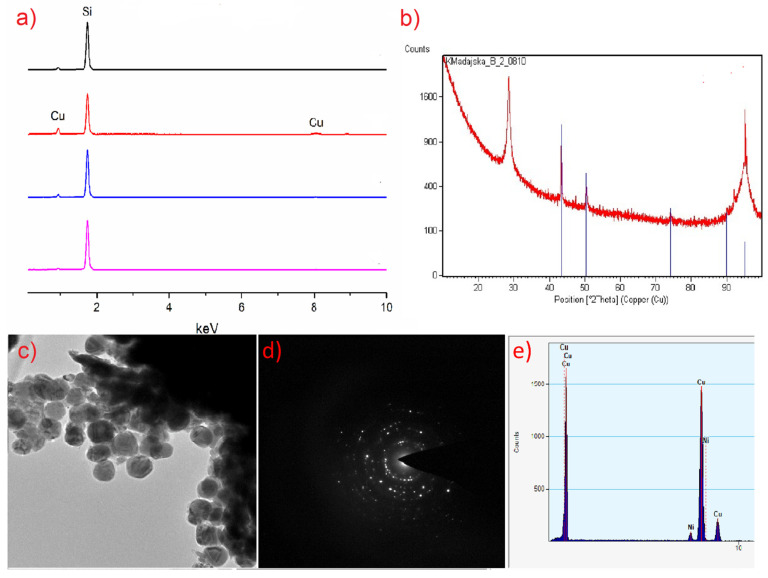
Composition analysis of the obtained layers using precursor **(2) [Cu_2_(AMDH)_2_(µ-O_2_CC_2_F_5_)_4_]**: (**a**) EDX spectra: black line: T_V_ = 453 K, T_D_ = 593 K; red, blue and pink line: T_V_ = 393 K, T_D_ = 633 K, in the case of the red line, the transport way of vapours was shorter; (**b**) XRD analysis; (**c**) TEM image; (**d**) diffraction pattern; (**e**) EDX spectra from TEM.

The deposited layer was smooth (Figure 14b and Figure 15b), and no impurity signals (C, N, O) were observed in the EDX spectrum (Figure 16a, red line).

In the case of compound (**2**), nanometric layers with a thickness of 69–360 nm were produced (Figure 14). It is seen that the higher evaporation temperature of the precursor results in a greater layer thickness (Figure 14a). This is consistent with the spectra of infrared spectroscopy because, at 393 K (Figure 8b), the concentration of the metal carriers in the gas phase was much lower than at 453 K (Figure 8b). Using the higher vaporization temperature (T_V_ = 453 K) and lower deposition temperature (T_D_ = 593 K) (Figure 14a), the grains (51–254 nm, Figure 15a) were more dispersed than at T_V_ = 393 K and T_D_ = 633 K (Figure 14b–d). The calculated roughness parameter R_a_ for the layer shown in Figure 14a was 51.0 nm, while for the films shown in Figure 14b–d, it was 11.1 nm, 11.1 nm, and 9.4 nm, respectively. This confirms that the layers deposited at lower vaporization and higher deposition temperature were smoother. The grain size for the films deposited at the higher temperatures was in the range of 36–201 nm (Figure 15c,d). Moreover, using a shorter transport way of the metal carriers, densely packed single grains with a size of 138–445 nm were obtained (Figure 15b).

**Figure 17:**
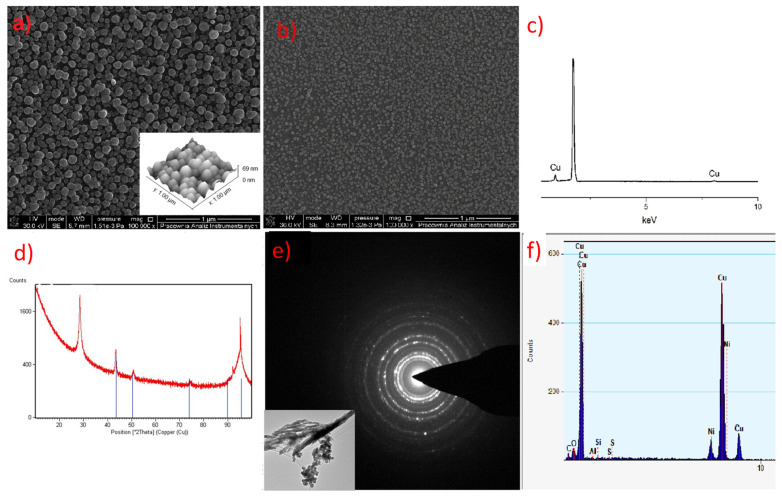
(**a**) SEM and AFM results of preliminary CVD experiments for the **[Cu_2_(AMDH)_2_(µ-O_2_CC_3_F_7_)_4_]** precursor (**3**) T_V_ = 453 K, T_D_ = 633 K; (**b**) SEM results, **[Cu_2_(AMDH)_2_(µ-O_2_CC_3_F_7_)_4_] (3),** T_V_ = 393 K, T_D_ = 573 K; (**c**) EDX spectra from SEM; (**d**) XRD spectra; (**e**) TEM image and diffraction pattern; (**f**) EDX spectra from TEM.

In the layer with a thickness of 69 nm deposited with complex (**3**), single, densely packed, round shape grains were also formed (Figure 17a). The continuity of the layer is also confirmed by the low value of the roughness parameter (R_a_ = 9.9 nm). It is worth noting that using a higher evaporation temperature T_V_ from 551 K (**3**) to 586 K for complex (**3**) leads to obtaining layers of similar thickness than when using a lower temperature for precursor (**2**). This also confirms the lower volatility of compound (**3**).

It is worth adding that both the precursor evaporation and decomposition temperatures were lower for the complex with perfluorinated amidine than for the previously used complex with ^t^BuNH_2_ [Cu_2_(^t^BuNH_2_)_2_(µ–O_2_CR_f_)_4_], which was 393 K, 453 K (amidine); 435–473 K (^t^BuNH_2_) and 573–633 K (amidine); 623–733 K (^t^BuNH_2_), respectively [42]. This shows that the introduction of a secondary perfluorinated ligand in the axial position increases the volatility of the compounds.

The EDX (SEM analysis) and XRD spectra of the layers showed that they contain metallic copper (Figure 16a,b and Figure 17b,c). The formation of metallic copper during the CVD process was also confirmed by the diffraction patterns and EDX spectra obtained by TEM analysis (Figure 16c and Figure 17d,e). The great advantage of compound (**2**) is that it was possible to obtain pure copper films from the air-stable copper(II) complex in an argon atmosphere without using an additional reducing agent. However, in the case of complex (**3**), apart from metallic copper, copper(I) oxide and a small amount of carbon (2.9 at.%) were also detected. In contrast, the layers deposited with the use of [Cu_2_(NH_2_^t^Bu)_2_(µ-O_2_CR_F_)_4_] precursors contained metallic copper and a low content of fluorine (1.5 at.%) [42]. Interestingly, despite the high content of fluorine in compounds (**2**) and (**3**), it is not present in the obtained deposits.

## 4. Conclusions

Copper(II) carboxylate compounds with perfluorinated amidine of the general formula [Cu_2_(AMDH)_2_(µ-O_2_CR_F_)_4_], where R_F_ = CF_3_, C_2_F_5_, C_3_F_7_, C_4_F_9_, were obtained by the reaction of copper(II) perfluorinated carboxylates with perfluorinated amidine. The IR spectra analysis suggested the bridging coordination mode of carboxylates. =NH-bonding of amidine was proposed from infrared spectra analysis and confirmed by DFT calculations. The structure of the obtained compounds was also confirmed by the results of the EI MS analysis. The following pseudomolecular ions [Cu_2_(AMDH)_2_(µ-O_2_CR_F_)_3_]^+^ proved the dimeric structure of the studied complexes with bridging carboxylates forming the “paddle-wheel” structure and axially N-coordinated amidines. The results of mass spectrometry (MS-EI) and variable temperature infrared spectroscopy demonstrated that the synthesised compounds are a source of copper carriers in the gas phase. Studies have shown that the decomposition process of the compounds is complicated. However, two main mechanisms can be distinguished: amidine deprotonation and formation of volatile copper amidinates and a carboxylic acid, and amidine detachment followed by decarboxylation of copper carboxylate. Moreover, the thermal analysis showed that copper transfer to the gaseous phase occurred even at atmospheric pressure.

The preliminary CVD experiments confirmed that compounds (**2**) and (**3**) are the source of metal carriers in the gas phase. Complex (**2**) was also shown to be the most volatile. Copper films were successfully deposited using precursor **[Cu_2_(AMDH)_2_(µ-O_2_CC_2_F_5_)_4_]** **(2)** by the CVD method in the Ar atmosphere. The great advantage of the **[Cu_2_(AMDH)_2_(µ-O_2_CC_2_F_5_)_4_] (2)** is that a metallic film can be obtained from the air-stable copper(II) compound without the need to use any additional reducing agent such as H_2_. The best results (smooth and pure structures) were achieved when the temperature of the precursor evaporation was 393 K, while the decomposition temperature was 633 K. Thanks to our research, we have obtained a promising compound that can be used as a precursor in CVD methods. Moreover, based on the study of the complexes’ interactions with high-energy electrons, it can be concluded that the energy and dose influence the deposit composition and compound **[Cu_2_(AMDH)_2_(µ-O_2_CC_2_F_5_)_4_] (2)** may also be a precursor in the FEBID method for the preparation of nanostructures composed of copper(II) fluoride.

## Figures and Tables

**Table 1:** Selected IR absorption bands (cm^−1^) of the studied compounds.

Compound	ν_asNH2_	ν_=NH_	δ_NH2_	ν_N=C–N_	ν_asCOO_	ν_sCOO_	Δ_COO_
**[Cu_2_(AMDH)_2_(µ-O_2_CCF_3_)_4_] (1)**	3380	3009	1597	1507	1649	1443	206
**[Cu_2_(AMDH)_2_(µ-O_2_CC_2_F_5_)_4_] (2)**	3390	3240	1603	1510	1657	1418	239
**[Cu_2_(AMDH)_2_(µ-O_2_CC_3_F_7_)_4_] (3)**	3387	3012	1606	1504	1675	1411	264
**[Cu_2_(AMDH)_2_(µ-O_2_CC_4_F_9_)_4_] (4)**	3390	3202	1608	1502	1672	1413	259
AMDH	3362	3130	1593	1450	-	-	-

Δν(CF_3_CO_2_Na) = 223 cm^−1^, Δν(C_2_F_5_CO_2_Na) = 268 cm^−1^, Δν(C_n_F_2n+1_CO_2_Na; n = 3–4) = 272 cm^−1^.

**Table 2:** Thermal analysis results.

Complex	Temperature (K)			Residue Cu_2_O (%)	
	T_i_	T_m_	T_f_	Found	Calc.
**[Cu_2_(AMDH)_2_(µ-O_2_CCF_3_)_4_] (1)**	391 544	471 573	492 586	13.0	15.8
**[Cu_2_(AMDH)_2_(µ-O_2_CC_2_F_5_)_4_] (2)**	364	449	556	3.59	13.0
**[Cu_2_(AMDH)_2_(µ-O_2_CC_3_F_7_)_4_] (3)**	337	499	551	1.96	11.0
**[Cu_2_(AMDH)_2_(µ-O_2_CC_4_F_9_)_4_] (4)**	338	513	555	4.64	9.52

T_i_—initial temperature, T_m_—maximum temperature, T_f_—final temperature.

**Table 3:** Mass spectrometry with electron ionisation (EI MS) results for **[Cu_2_(AMDH)_2_(µ-O_2_C_2_F_5_)_4_] (2)**.

Fragments	m/z	Temperature [K]				
		327	353	398	433	529
[Cu_2_(AMDH)_2_(O_2_CC_2_F_5_)_3_]^+^	939	8	24	7	3	-
[Cu_2_(AMDH)(HN=C=NH)(O_2_CC_2_F_5_)_3_]^+^	819	<1	2	<1	-	-
[Cu_2_(AMDH)_2_(O_2_CC_2_F_5_)_2_]^+^	776	1	3	4	7	-
[Cu_2_(AMD)_2_(O_2_CC_2_F_5_)_2_]^+.^	774	<1	1	1	-	-
[Cu_2_(AMD)_3_]^+^	609	2	7	2	1	-
[Cu(AMDH)(AMD)(O_2_CC_2_F_5_)]^+^	549	9	30	10	4	-
[Cu_2_(O_2_CC_2_F_5_)_2_]^+.^	452	1	4	3	3	18
[Cu_2_(AMDH)(O_2_CC_2_F_5_)]^+^	451	2	5	3	4	<1
[Cu_2_(AMD)(O_2_CC_2_F_5_)]^+^	450	2	4	4	8	<1
[Cu(AMDH)_2_]^+^	387	4	14	9	-	-
[Cu(AMD)_2_]^+.^	385	6	18	7	-	-
[Cu_2_(O_2_CC_2_F_5_)]^+^	289	2	5	5	10	50
[Cu(AMDH)(HN=C=NH)]^+^	267	4	10	6	3	-
[Cu(AMD)(HN=C=NH)]^+^	266	1	3	1	1	-
[AMDH]^+.^	162	9	39	7	8	-
[NH_2_CC_2_F_5_]^+^	147	9	34	11	23	12
[Cu_2_F]^+^	145	-	<1	1	2	13
[C_2_F_4_CN]^+^	126	62	100	66	75	51
[C_2_F_5_]^+^	119	86	1	100	1	100
[CF_2_CN]^+^	76	80	1	85	100	67
[Cu]^+^	63	-	1	2	2	9
[CO_2_H]^+^	45	40	1	45	90	24
[CO_2_]^+.^	44	100	75	76	90	1
[HN=C=NH]^+.^	42	5	7	2	2	2
[F_2_]^+.^	38	9	3	3	2	1

**Table 4:** Summary of chemical vapour deposition (CVD) conditions for the compounds (**2**) and (**3**).

Precursor	
Precursor mass (m) [mg]	100
Vaporization temperature (T_V_) [K]	393 (**2**), 453 (**2**), (**3**)
Deposition temperature (T_D_) [K]	573–633
Carrier gas	**Ar**
Substrates	Si(111)
Deposition time (t) [min]	60

## Data Availability

The data presented in this study are available on request from the corresponding author.

## Supplementary Materials

The following are available online at https://www.mdpi.com/article/10.3390/ma14123145/s1, Figure S1. Infrared spectra for the complex [Cu_2_(AMDH)_2_(µ-O_2_CF_3_)_4_] (1); Figure S2. Infrared spectra for the complex [Cu_2_(AMDH)_2_(µ-O_2_C_2_F_5_)_4_] (2); Figure S3. Infrared spectra for the complex [Cu_2_(AMDH)_2_(µ-O_2_C_3_F_7_)_4_] (3); Figure S4. Infrared spectra for the complex [Cu_2_(AMDH)_2_(µ-O_2_C_4_F_9_)_4_] (4); Figure S5. VT IR spectra of [Cu_2_(AMDH)_2_(µ-O_2_CCF_3_)_4_] (1) in the temperature range 333–753 K; Figure S6. VT IR spectra of [Cu_2_(AMDH)_2_(µ-O_2_CC_3_F_7_)_4_] (3) in the temperature range 333–613 K; Figure S7. VT IR spectra of [Cu_2_(AMDH)_2_(µ-O_2_CC_4_F_9_)_4_] (4) in the temperature range 333–633 K; Figure S8. IR spectrum in the gas phase at 353 K for the complex [Cu_2_(AMDH)_2_(µ-O_2_CC_2_F_5_)_4_] (2); Table S1. EI MS results for the complex [Cu_2_(AMDH)_2_(µ-O_2_CF_3_)_4_] (1); Table S2. EI MS results for the complex [Cu_2_(AMDH)_2_(µ-O_2_C_3_F_7_)_4_] (3); Table S3. EI MS results for the complex [Cu_2_(AMDH)_2_(µ-O_2_C_4_F_9_)_4_] (4).
