# Supplementary material for: New Volatile Perfluorinated Amidine–Carboxylate Copper(II) Complexes as Promising Precursors in CVD and FEBID Methods
Source: Materials (Basel). 2021 Jun 8;14(12):3145. doi: 10.3390/ma14123145 (PMC8230148; doi:10.3390/ma14123145)
Supplement: Supplementary file 1 [file materials-14-03145-s001.zip › materials-1216738-supplementary.pdf]

Supplementary Materials

New Volatile Perfluorinated Amidine–Carboxylate Copper(II) Complexes as Promising Precursors in CVD and FEBID Methods

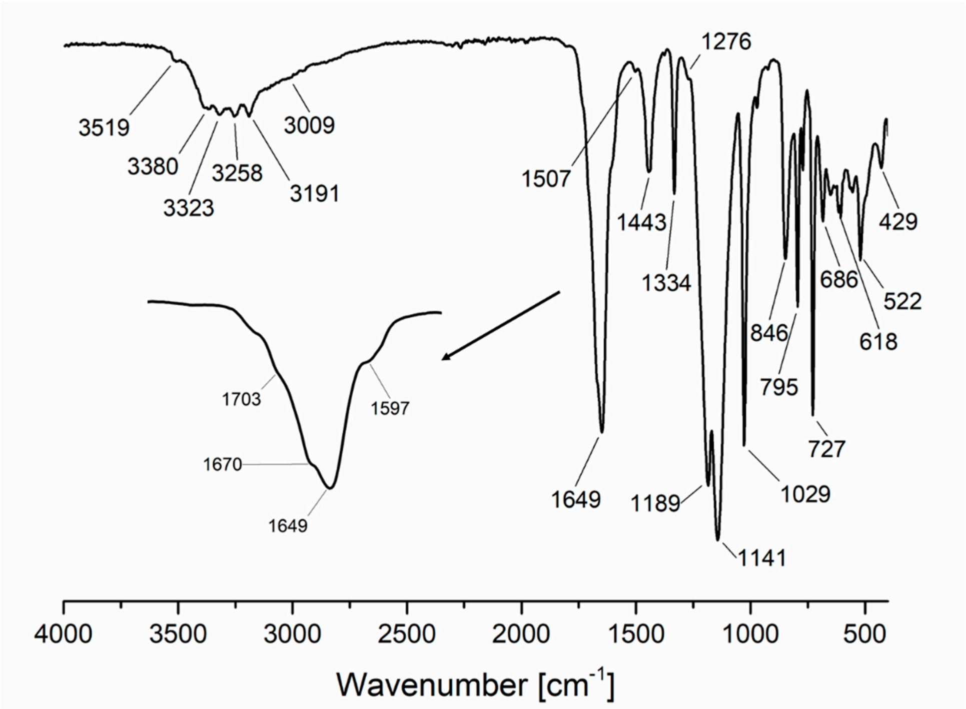

Figure S1. Infrared spectra for the complex **[Cu_2(AMDH)$_2$(μ-O_2CF_3)$_4$] (1)**.

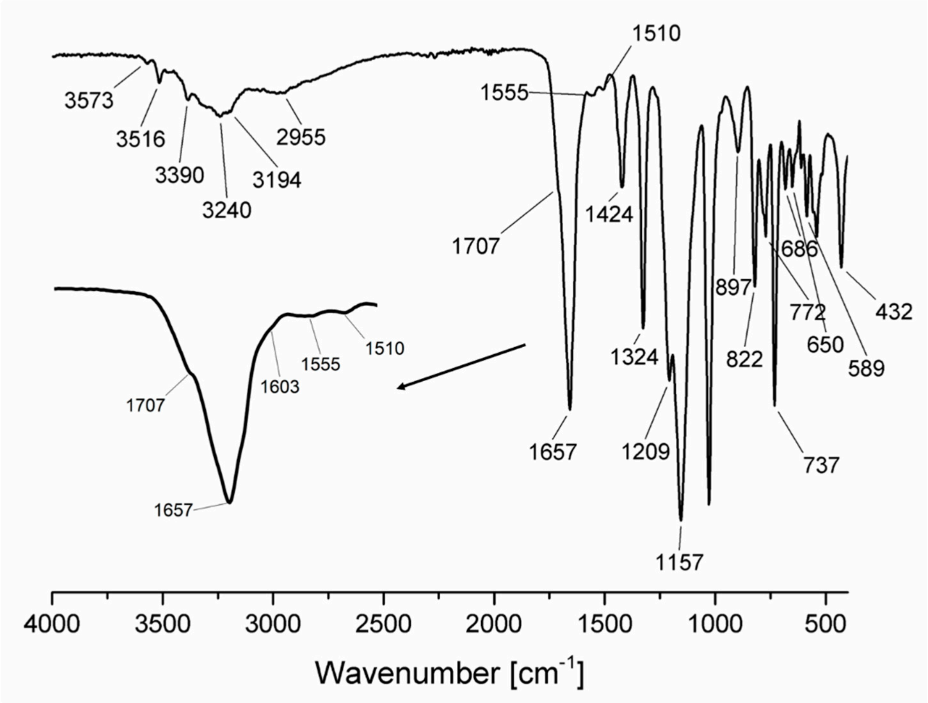

Figure S2. Infrared spectra for the complex **[Cu_2(AMDH)$_2$(μ-$O_2C_2F_5$)$_4$] (2)**.

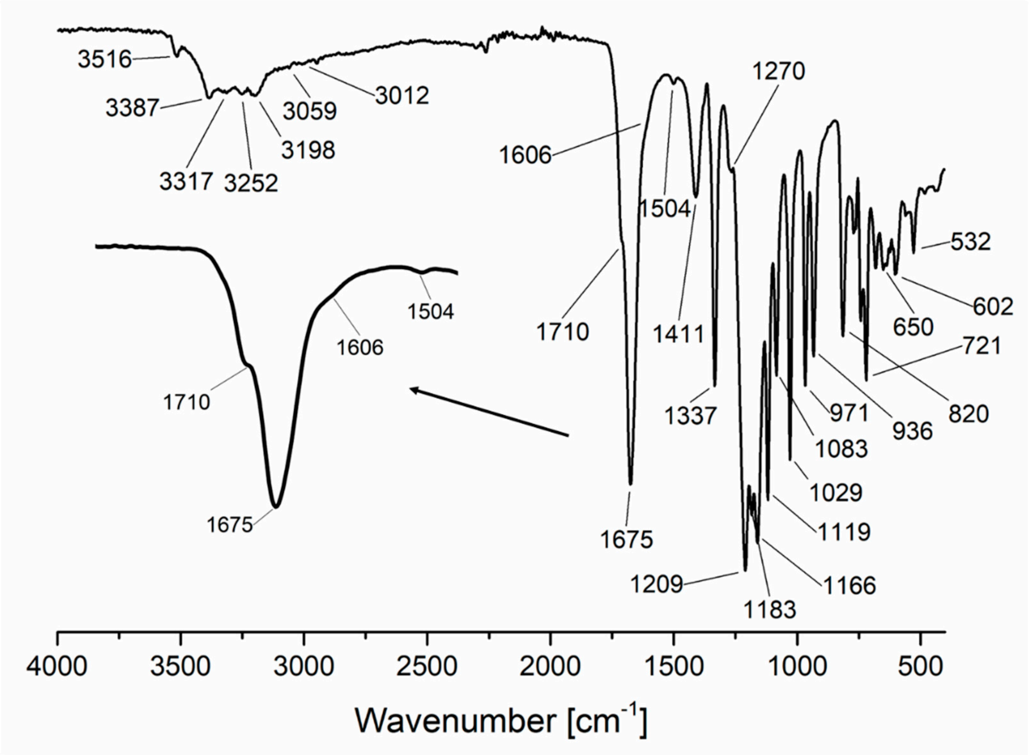

Figure S3. Infrared spectra for the complex **[Cu_2(AMDH)$_2$(μ-$O_2C_3F_7$)$_4$] (3)**.

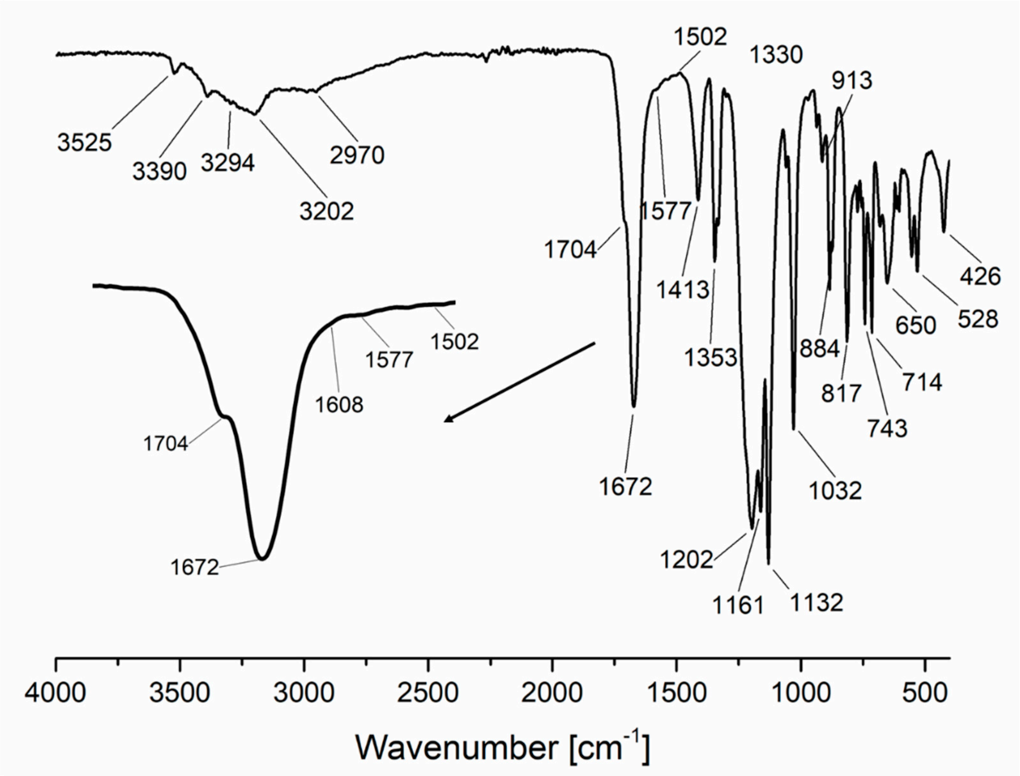

Figure S4. Infrared spectra for the complex **[Cu_2(AMDH)$_2$(μ-$O_2C_4F_9$)$_4$] (4)**.

Table S1. EI MS results for the complex **$[Cu_2(AMDH)_2(\mu\text{-}O_2CF_3)_4]$ (1)**.

Fragments	m/z	Temperature [K]				
		313	357	396	426	538
**$[Cu_2(AMDH)_2(O_2CCF_3)_3]^+$**	789	4	13	67	2	-
**$[Cu_2(AMDH)_2(O_2CCF_3)_2]^{+\cdot}$**	676	<1	1	6	1	-
**$[Cu_2(AMD)_2(O_2CCF_3)_2]^{+\cdot}$**	674	<1	1	6	1	-
**$[Cu_2(NH_2)(AMDH)_2(AMD)]^+$**	627	1	2	14	4	-
**$[Cu_2(NH_2)(AMD)_2(O_2CCF_3)]^{+\cdot}$**	577	-	<1	1	5	-
**$[Cu_2(AMD)_2(O_2CCF_3)\text{-}H]^+$**	560	1	4	23	4	-
**$[Cu(AMDH)_2(O_2CCF_3)]^+$**	500	6	16	82	3	-
**$[Cu_2(AMD)_2]^{+\cdot}$**	448	1	3	29	12	-
**$[Cu_2(AMDH)(O_2CCF_3)]^+$**	401	2	3	21	5	2
**$[Cu_2(AMD)(O_2CCF_3)]^{+\cdot}$**	400	1	3	38	12	1
**$[Cu(AMDH)_2]^+$**	387	3	6	30	2	<1
**$[Cu(AMDH)(AMD)]^+$**	386	6	14	71	5	<1
**$[Cu_2(O_2CCF_3)_2]^{+\cdot}$**	352	<1	1	6	2	38
**$[Cu(AMDH)(O_2CCF_3)]^+$**	338	2	5	28	8	<1
**$[Cu_2(AMD)]^+$**	287	1	3	33	10	2
**$[Cu(AMDH)(HN{=}C{=}NH)]^+$**	267	6	10	39	7	<1
**$[Cu(AMD)(HN{=}C{=}NH)]^+$**	266	2	3	12	3	1
**$[Cu(AMD)(HCN)]^+$**	251	<1	1	3	22	<1
**$[Cu_2(O_2CCF_3)]^+$**	239	1	4	27	11	74
**$[Cu(AMDH)]^+$**	225	11	24	100	46	<1
**$[AMDH]^+$**	162	55	41	1	84	<1
**$[C_2F_5CNH]^+$**	146	9	7	18	14	1
**$[Cu_2F]^+$**	145	1	1	7	4	23
**$[C_2F_4CNH]^+$**	127	12	8	18	19	4
**$[C_2F_4CN]^+$**	126	1	1	1	2	100
**$[C_2F_5]^+$**	119	1	100	1	1	60
**$[C_2F_4]^{+\cdot}$**	100	<1	52	95	100	36
**$[CO_2]^{+\cdot}$**	44	7	2	1	1	6
**$[HN{=}C{=}NH]^{+\cdot}$**	42	48	23	60	42	12
**$[F_2]^{+\cdot}$**	38	100	18	10	14	10

Table S2. EI MS results for the complex **[$Cu_2(AMDH)_2(\mu\text{-}O_2C_3F_7)_4$] (3)**.

Fragment	m/z	Temperature [K]				
		358	383	435	486	550
**[$Cu_3(O_2CC_3F_7)_5$]$^+$**	1256	-	-	1	5	1
**[$Cu_3(AMD)(O_2CC_3F_7)_4$]$^+$**	1204	-	-	1	4	-
**[$Cu_2(AMDH)_2(O_2CC_3F_7)_3$]$^+$**	1089	2	2	<1	<1	-
**[$Cu_2(AMDH)(O_2CC_3F_7)_3$]$^+$**	927	<1	2	2	3	-
**[$Cu_2(AMDH)(AMD)(O_2CC_3F_7)_2$]$^+$**	875	-	<1	-	-	-
**[$Cu_2(AMDH)(O_2CC_3F_7)$]$^+$**	714	-	<1	<1	1	-
**[$Cu_2(AMD)(O_2CC_3F_7)_3$]$^+$**	713	-	<1	<1	2	-
**[$Cu_2(AMD)_2(O_2CC_3F_7)$ - H]$^+$**	660	<1	<1	<1	1	-
**[$Cu(AMDH)_2(O_2CC_3F_7)$]$^+$**	600	2	3	1	<1	-
**[$Cu_2(O_2CC_3F_7)_2$]$^+$**	552	-	-	2	20	96
**[$Cu_2(AMDH)(O_2CC_3F_7)$]$^+$**	501	<1	1	3	6	<1
**[$Cu_2(AMD)(O_2CC_3F_7)$]$^+$**	500	<1	<1	5	34	<1
**[$Cu_2(AMDH)_2$]$^+$**	450	<1	<1	2	7	<1
**[$Cu_2(AMD)_2$]$^+$**	448	<1	<1	2	7	<1
**[$Cu(AMDH)(O_2CC_3F_7)$]$^+$**	438	<1	1	3	4	-
**[$Cu(AMDH)(AMD)$]$^+$**	386	1	2	<1	<1	<1
**[$Cu(AMD)_2$]$^+$**	385	-	-	<1	2	5
**[$Cu_3(NH_2)_2(AMD)$ - H]$^+$**	383	-	<1	<1	3	4
**[$Cu_2(O_2CC_3F_7)$]$^+$**	339	-	<1	7	69	2
**[$Cu_2(AMD)$]$^+$**	287	<1	1	5	52	1
**[$Cu(HCN)(AMD)$]$^+$**	251	-	-	<1	20	<1
**[$Cu(AMDH)$]$^+$**	225	4	2	7	13	<1
**[C_3F_7]$^+$**	169	18	26	37	1	1
**[AMDH]$^{+\cdot}$**	162	13	15	7	13	<1
**[Cu_2F]$^+$**	145	-	<1	13	18	97
**[C_2F_4CN]$^+$**	126	1	1	100	3	17
**[C_2F_5]$^+$**	119	1	1	1	2	1
**[C_2F_4]$^+$**	100	100	1	1	1	4
**[CF_2CN]$^+$**	76	1	1	1	1	50
**[CO_2H]$^+$**	45	30	42	34	100	47
**[CO_2]$^+$**	44	3	3	2	2	4
**[HN=C=NH]$^{+\cdot}$**	42	14	11	7	8	2
**[N≡C-NH]$^+$**	41	1	100	55	19	1
**[F_2]$^{+\cdot}$**	38	37	19	9	9	1

Table S3. EI MS results for the complex **$[Cu_2(AMDH)_2(\mu\text{-}O_2C_4F_9)_4]$ (4)**.

Fragment	m/z	Temperature [K]				
		371	396	428	460	505
**$[Cu_3(AMDH)(O_2CC_4F_9)_5]^+$**	1668	-	<1	3	3	-
**$[Cu_3(O_2CC_4F_9)_5]^+$**	1506	-	-	2	14	16
**$[Cu_3(AMD)(O_2CC_4F_9)_4]^+$**	1404	-	-	2	17	4
**$[Cu_4(NH_2)_2(AMDH)(AMD)_2(O_2CC_4F_9)_2]^{+\cdot}$**	1296	-	-	1	4	<1
**$[Cu_2(AMDH)_2(O_2CC_4F_9)_3]^+$**	1239	6	38	35	5	1
**$[Cu_2(AMDH)(O_2CC_4F_9)_3]^+$**	1077	2	8	33	23	2
**$[Cu_3(NH_2)(O_2CC_4F_9)_3]^+$**	996	2	<1	2	4	2
**$[Cu_3(O_2CC_4F_9)_3]^{+\cdot}$**	980	-	<1	1	4	3
**$[Cu_2(AMDH)(AMD)(O_2CC_4F_9)_2]^{+\cdot}$**	975	<1	2	3	1	<1
**$[Cu_2(O_2CC_4F_9)_3]^+$**	915	-	<1	1	2	4
**$[Cu_3(NH_2)(AMD)(O_2CC_4F_9)_2]^+$**	894	-	<1	2	5	3
**$[Cu_3(NH_2)(AMD)_2(O_2CC_4F_9)]^+$**	792	-	<1	1	4	1
**$[Cu_3(O_2CC_4F_9)_2]^+$**	717	-	<1	1	6	5
**$[Cu_2(AMD)_2(O_2CC_4F_9) - H]^+$**	710	1	7	9	7	1
**$[Cu_2(O_2CC_4F_9)_2]^{+\cdot}$**	652	3	23	35	43	53
**$[Cu(AMDH)_2(O_2CC_4F_9)]^+$**	650	6	47	49	7	<1
**$[Cu_2(AMDH)(O_2CC_4F_9)]^+$**	551	1	10	21	23	7
**$[Cu_2(AMD)(O_2CC_4F_9)]^{+\cdot}$**	550	1	7	26	84	44
**$[Cu(AMDH)(HN{=}C{=}NH)(O_2CC_4F_9)]^+$**	530	<1	4	4	1	<1
**$[Cu(AMDH)(O_2CC_4F_9)]^+$**	488	1	8	27	23	2
**$[Cu_2(AMD)_2]^{+\cdot}$**	448	1	4	7	17	7
**$[Cu_2(O_2C)(O_2CC_4F_9)]^{+\cdot}$**	433	<1	1	3	9	7
**$[Cu_2(O_2CC_4F_9)]^+$**	389	1	21	75	1	1
**$[Cu_2(AMD)]^+$**	287	1	9	29	68	43
**$[Cu(AMDH)(HN{=}C{=}NH)]^+$**	267	4	21	13	4	1
**$[Cu(AMD)(HCN)]^+$**	251	<1	1	1	3	70
**$[Cu(AMDH)]^+$**	225	6	54	99	57	7
**$[C_4F_9]^+$**	219	2	16	52	100	100
**$[AMDH]^{+\cdot}$**	162	5	23	43	31	14
**$[Cu_2F]^+$**	145	<1	3	18	34	41
**$[C_2F_4CN]^+$**	126	1	1	2	4	6
**$[C_2F_5]^+$**	119	100	1	1	1	2
**$[C_2F_4]^+$**	100	81	1	2	3	4
**$[CF_2CN]^+$**	76	1	1	1	1	1
**$[CF_2]^+$**	50	69	88	100	75	89
**$[CO_2H]^+$**	45	16	67	1	1	1
**$[CO_2]^+$**	44	3	3	3	2	3
**$[HN{=}C\text{-}NH_2]^{+\cdot}$**	43	35	100	1	84	24
**$[HN{=}C{=}NH]^{+\cdot}$**	42	15	16	19	9	4
**$[F_2]^{+\cdot}$**	38	59	29	10	4	4

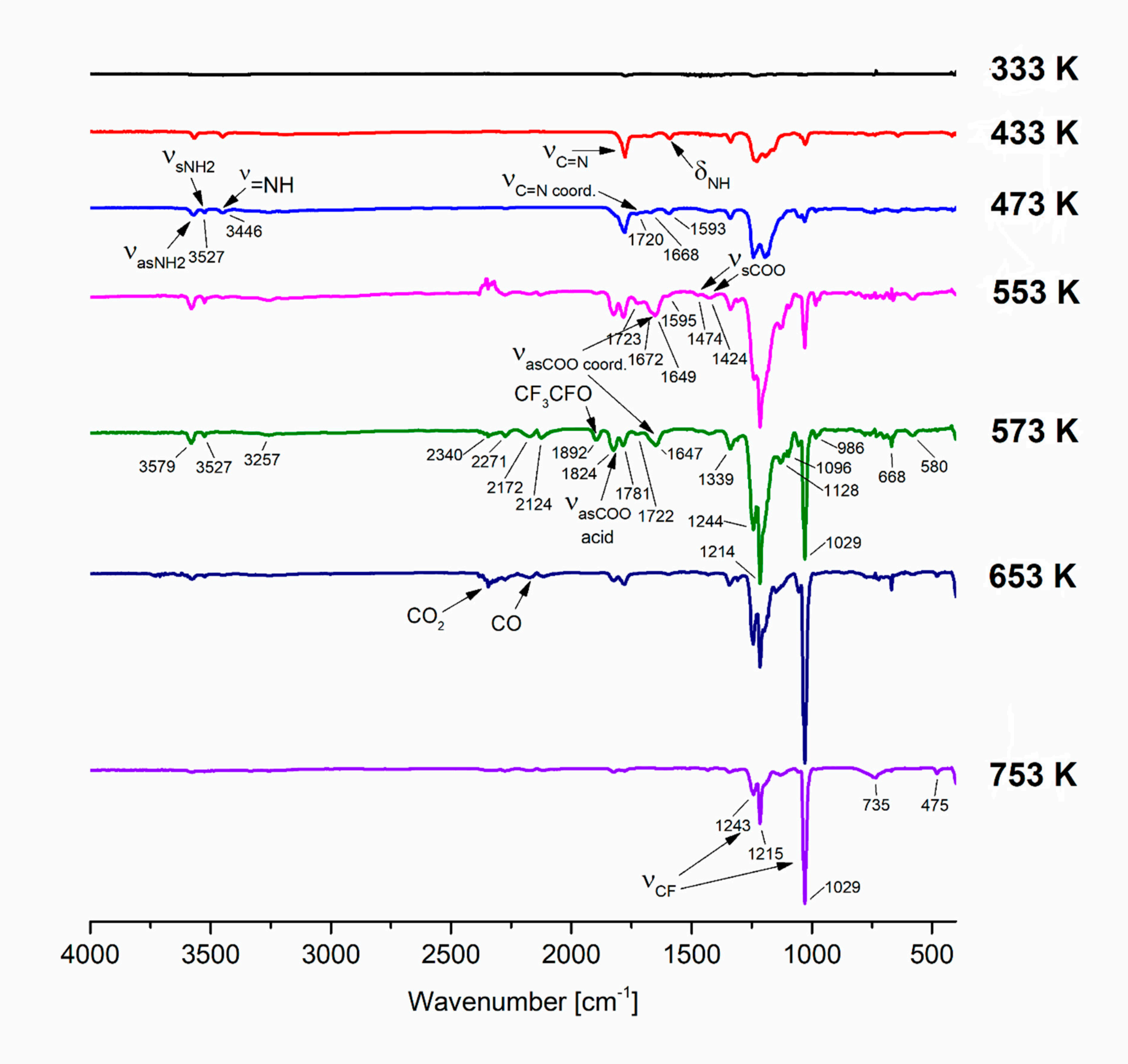

Figure S5. VT IR spectra of **[Cu$_2$(AMDH)$_2$(μ-O$_2$CCF$_3$)$_4$] (1)** in the temperature range 333–753 K.

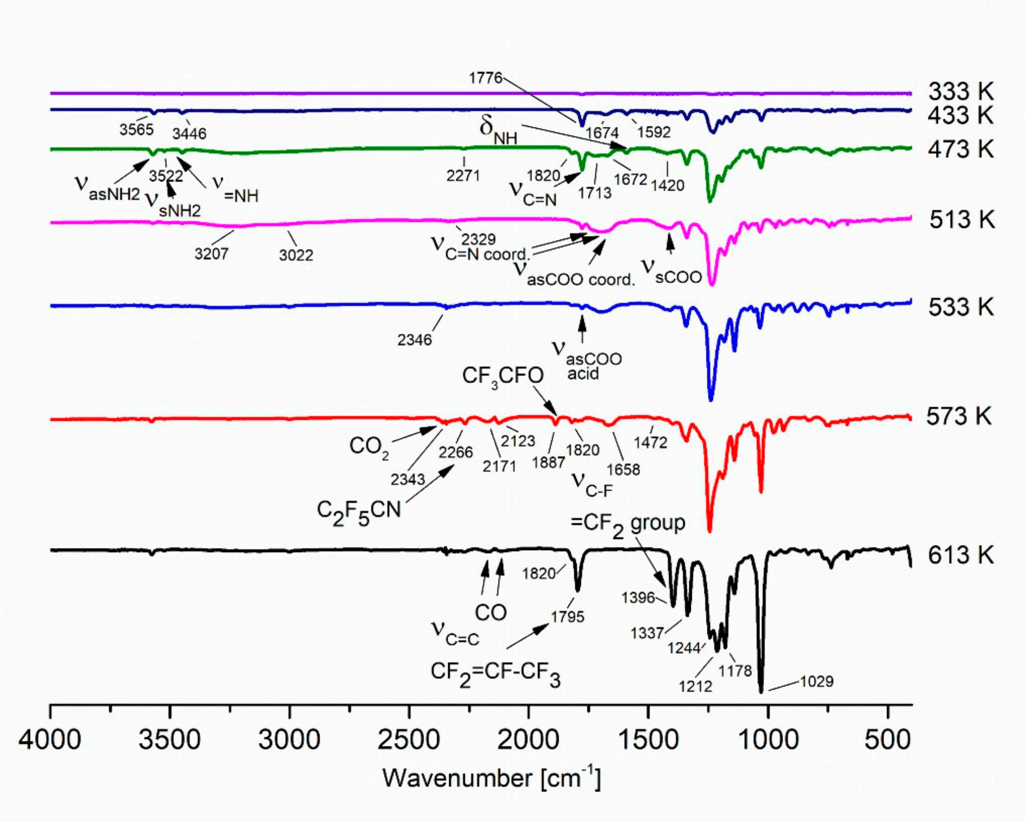

Figure S6. VT IR spectra of **[Cu$_2$(AMDH)$_2$(μ-O$_2$CC$_3$F$_7$)$_4$] (3)** in the temperature range 333–613 K.

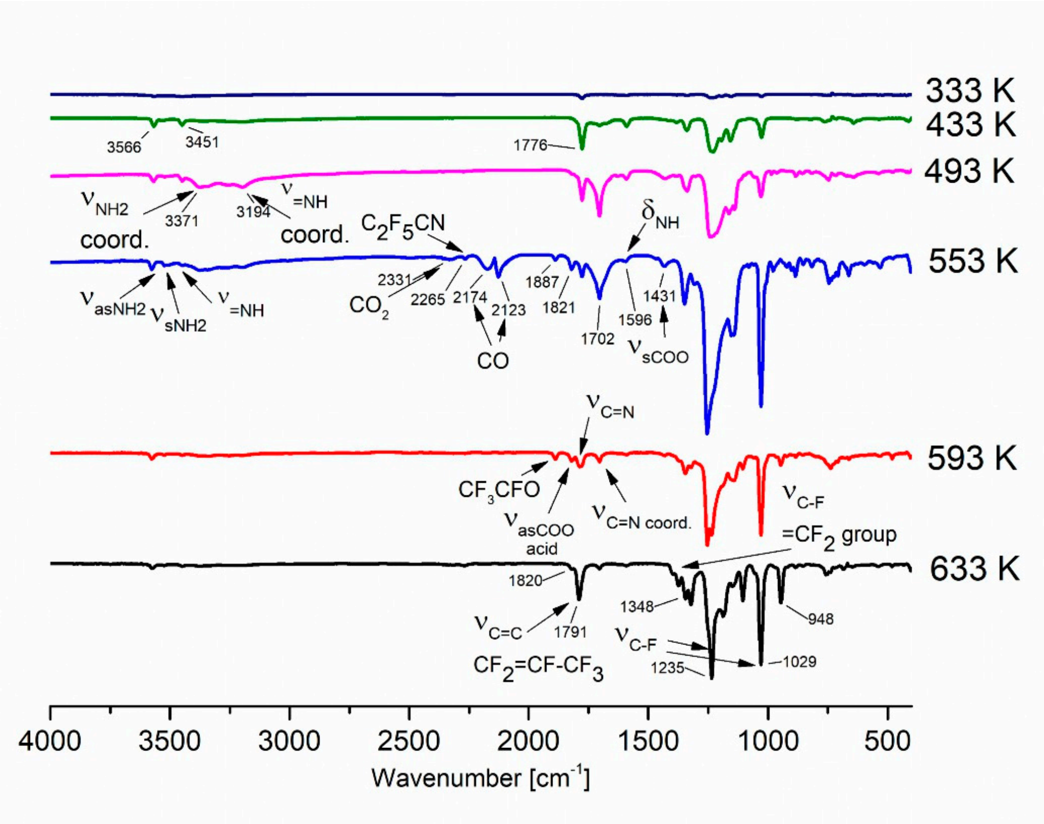

Figure S7. VT IR spectra of **[Cu$_2$(AMDH)$_2$(μ-O$_2$CC$_4$F$_9$)$_4$] (4)** in the temperature range 333–633 K.

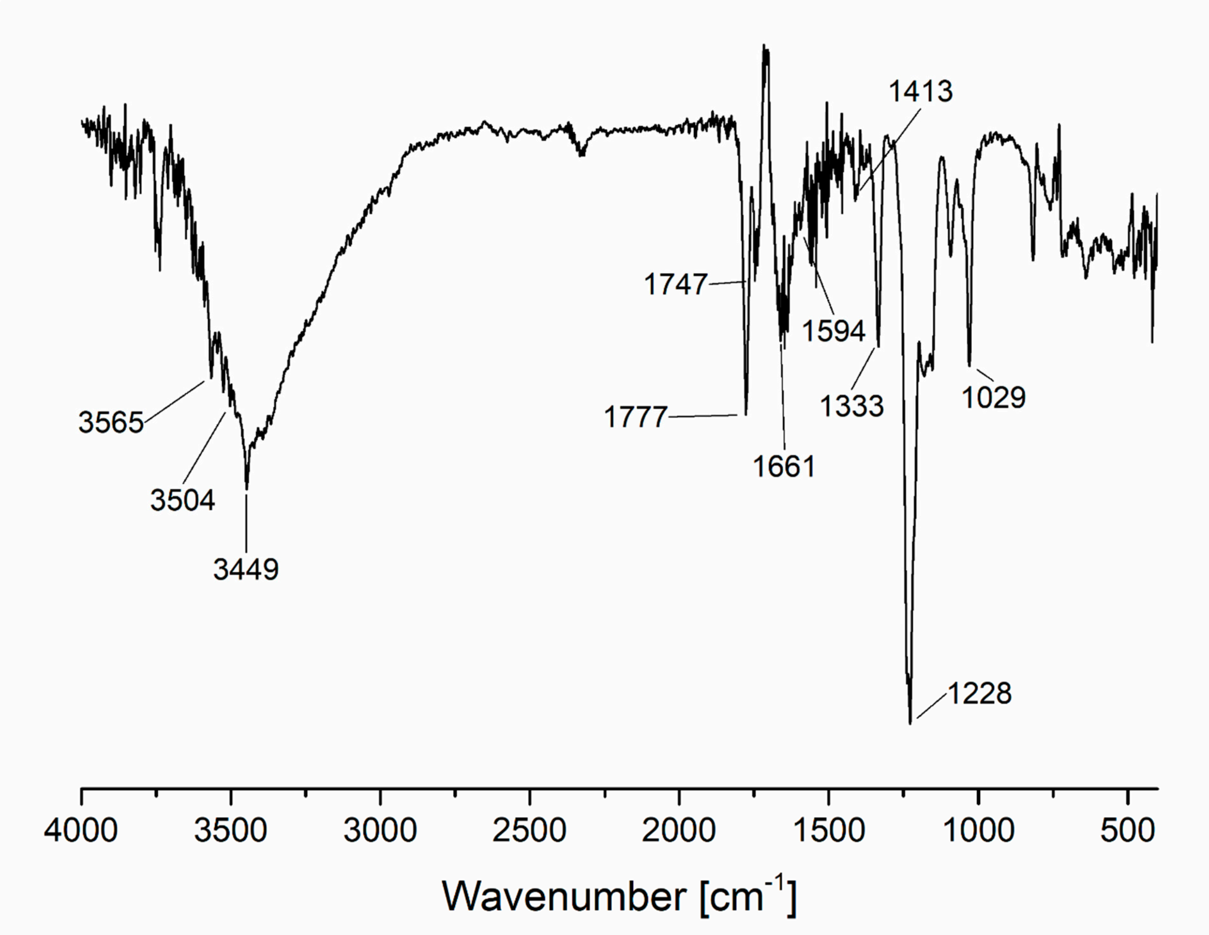

Figure S8. IR spectrum in the gas phase at 353 K for the complex **[$Cu_2(AMDH)_2(\mu\text{-}O_2CC_2F_5)_4$] (2)**.
